# Supplementary material for: Associations between Blood Metabolic Profile at 7 Years Old and Eating Disorders in Adolescence: Findings from the Avon Longitudinal Study of Parents and Children
Source: Metabolites. 2019 Sep 19;9(9):191. doi: 10.3390/metabo9090191 (PMC6780115; doi:10.3390/metabo9090191)
Supplement: Supplementary file 1 [file metabolites-09-00191-s001.zip › metabolites-562545-SupplementaryMaterial.docx]

**Supplementary data**

Associations between blood metabolome at 7 years old and eating disorders in adolescence: findings from Avon Longitudinal Study of Parents and Children cohort

Diana L. Santos Ferreira^1, 2#^, Christopher Hübel^3,4,5#^, Moritz Herle^6^, Mohamed Abdulkadir^7^, Ruth J. F. Loos^8^, Rachel Bryant-Waugh^6^, Cynthia M. Bulik^5,9,10^, Bianca L. De Stavola^6^, Deborah A. Lawlor^1, 2,11^ and Nadia Micali^6,7,12, *^

^1^ Medical Research Council Integrative Epidemiology Unit at the University of Bristol, Bristol BS8 2BN, UK; diana.santosferreira@bristol.ac.uk (D.L.S.F.); d.a.lawlor@bristol.ac.uk (D.A.L.)

^2^ Population Health Sciences, Bristol Medical School, University of Bristol, Bristol BS8 2PS, UK;

^3^ Social, Genetic & Developmental Psychiatry Centre, Institute of Psychiatry, Psychology & Neuroscience, King’s College London, London, SE5 8AF, UK; christopher.huebel@kcl.ac.uk (C.H.)

^4^ UK National Institute for Health Research (NIHR) Biomedical Research Centre, South London and Maudsley Hospital, London, SE5 8AF, UK;

^5^ Department of Medical Epidemiology and Biostatistics, Karolinska Institutet, Stockholm, Sweden, SE-171 77; cynthia_bulik@med.unc.edu (C.M.B.)

^6^ University College London, Great Ormond Street Institute of Child Health, London, WC1N 1EH, UK;

moritz.herle.12@ucl.ac.uk (M.H.); r.bryant-waugh@ucl.ac.uk (R.BW.); [b.destavola@ucl.ac.uk](mailto:b.destavola@ucl.ac.uk) (B.L.D.S.); n.micali@ucl.ac.uk (N.M.)

^7^ Department of Psychiatry, Faculty of Medicine, University of Geneva, Geneva, CH – 1205, Switzerland; mohamed.abdulkadir@unige.ch (M.A.)

^8^ Icahn Mount Sinai School of Medicine, New York, NY, USA; ruth.loos@mssm.edu (R.J.F.L.)

^9^ Department of Psychiatry, University of North Carolina at Chapel Hill, Chapel Hill, NC, USA; 27599

^10^ Department of Nutrition, University of North Carolina at Chapel Hill, Chapel Hill, NC, USA; 27599

^11^ Bristol National Institute of Health Research Biomedical Research Centre, Bristol BS1 3NU, UK;

^12^ Child and Adolescent Psychiatry Division, Department of Child and Adolescent Health, Geneva University Hospital, Geneva, CH – 1205, Switzerland;

***** Correspondence: n.micali@ucl.ac.uk; Tel.: +44-207-905-2163

^#^ Authors contributed equally

Table of Contents

[Supplementary figures 3](#_Toc16273107)

[Figure S1a. Estimated odds ratios for anorexia nervosa (AN) at 14, 16, 18 years of age and cumulatively across all three time points according to lipoprotein subclasses particle and lipid concentrations at 7 years. 3](#_Toc16273108)

[Figure S1b. Estimated odds ratios for anorexia nervosa (AN) at 14, 16, 18 years of age and cumulatively across all three time points according to lipoprotein subclasses particle and lipid concentrations at 7 years. 4](#_Toc16273109)

[Figure S1c. Estimated odds ratios for anorexia nervosa (AN) at 14, 16, 18 years of age and cumulatively across all three time points according to lipoprotein subclasses particle and lipid concentrations at 7 years. 5](#_Toc16273110)

[Figure S2a. Estimated odds ratios for anorexia nervosa (AN) at 14 years of age according to metabolic traits concentrations at 7 years. 6](#_Toc16273111)

[Figure S2b. Estimated odds ratios for anorexia nervosa (AN) at 14 years of age according to metabolic traits concentrations at 7 years. 7](#_Toc16273112)

[Figure S3a. Estimated odds ratios for anorexia nervosa (AN) at 16 years of age according to metabolic traits concentrations at 7 years. 8](#_Toc16273113)

[Figure S3b. Estimated odds ratios for anorexia nervosa (AN) at 16 years of age according to metabolic traits concentrations at 7 years. 9](#_Toc16273114)

[Figure S4a. Estimated odds ratios for anorexia nervosa (AN) at 18 years of age according to metabolic traits concentrations at 7 years. 10](#_Toc16273115)

[Figure S4b. Estimated odds ratios for anorexia nervosa (AN) at 18 years of age according to metabolic traits concentrations at 7 years. 11](#_Toc16273116)

[Figure S5a. Estimated odds ratios for anorexia nervosa (AN) by 18 years of age (i.e. cumulatively across 14, 16, 18 years old) according to metabolic traits concentrations at 7 years. 12](#_Toc16273117)

[Figure S5b. Estimated odds ratios for anorexia nervosa (AN) by 18 years of age (i.e. cumulatively across 14, 16, 18 years old) according to metabolic traits concentrations at 7 years. 13](#_Toc16273118)

[Figure S6a. Estimated odds ratios for binge-eating disorder (BED) at 14, 16, 18 years of age and cumulatively across all three time points according to lipoprotein subclasses particle and lipid concentrations at 7 years. 14](#_Toc16273119)

[Figure S6b. Estimated odds ratios for binge-eating disorder (BED) at 14, 16, 18 years of age and cumulatively across all three time points according to lipoprotein subclasses particle and lipid concentrations at 7 years. 15](#_Toc16273120)

[Figure S6c. Estimated odds ratios for binge-eating disorder (BED) at 14, 16, 18 years of age and cumulatively across all three time points according to lipoprotein subclasses particle and lipid concentrations at 7 years. 16](#_Toc16273121)

[Figure S7a. Estimated odds ratios for binge-eating disorder (BED) at 14 years of age according to metabolic traits concentrations at 7 years. 17](#_Toc16273122)

[Figure S7b. Estimated odds ratios for binge-eating disorder (BED) at 14 years of age according to metabolic traits concentrations at 7 years. 18](#_Toc16273123)

[Figure S8a. Estimated odds ratios for binge-eating disorder (BED) at 16 years of age according to metabolic traits concentrations at 7 years. 19](#_Toc16273124)

[Figure S8b. Estimated odds ratios for binge-eating disorder (BED) at 16 years of age according to metabolic traits concentrations at 7 years. 20](#_Toc16273125)

[Figure S9a. Estimated odds ratios for binge-eating disorder (BED) at 18 years of age according to metabolic traits concentrations at 7 years. 21](#_Toc16273126)

[Figure S9b. Estimated odds ratios for binge-eating disorder (BED) at 18 years of age according to metabolic traits concentrations at 7 years. 22](#_Toc16273127)

[Figure S10a. Estimated odds ratios for binge-eating disorder (BED) by 18 years of age (i.e. cumulatively across 14, 16, 18 years old) according to metabolic traits concentrations at 7 years. 23](#_Toc16273128)

[Figure S10b. Estimated odds ratios for binge-eating disorder (BED) by 18 years of age (i.e. cumulatively across 14, 16, 18 years old) according to metabolic traits concentrations at 7 years. 24](#_Toc16273129)

[Figure S11a. Estimated odds ratios for binge-eating disorder (BED) and anorexia nervosa (AN) at 14, 16, 18 years of age and cumulatively across all three time points according to metabolic traits concentration at 7 years. 25](#_Toc16273130)

[Figure S11b. Estimated odds ratios for binge-eating disorder (BED) and anorexia nervosa (AN) at 14, 16, 18 years of age and cumulatively across all three time points according to metabolic traits concentration at 7 years. 26](#_Toc16273131)

[Figure S11c. Odds Ratios for binge-eating disorder (BED) and anorexia nervosa (AN) at 14, 16, 18 years of age and cumulatively across all three time points according to metabolic traits concentration at 7 years. 27](#_Toc16273132)

[Figure S11d. Odds Ratios for binge-eating disorder (BED) and anorexia nervosa (AN) at 14, 16, 18 years of age and cumulatively across all three time points according to metabolic traits concentration at 7 years. 28](#_Toc16273133)

[Supplementary tables 29](#_Toc16273134)

[Table S1. Distribution of background characteristics according to follow-up information available in the Avon Longitudinal Study of Parents and Children (ALSPAC) cohort. 29](#_Toc16273135)

# Supplementary figures

Figure S1a. Estimated odds ratios for anorexia nervosa (AN) at 14, 16, 18 years of age and cumulatively across all three time points according to lipoprotein subclasses particle and lipid concentrations at 7 years. Estimates refer to 1 standard deviation increase in metabolic trait concentration at 7 years. Error bars = 95% confidence intervals (CI). Abbreviations: C=cholesterol; IDL=intermediate-density lipoprotein; LDL=low-density lipoprotein; HDL=high-density lipoprotein; MUFA=monounsaturated fatty acids; PUFA=polyunsaturated fatty acids; VLDL=very-low-density lipoprotein. Note: Filled dot: CI do not include the null. MUFA, PUFA and saturated fatty acid concentrations include all fatty acids detected which have one, more than one, or zero C=C double bonds in their backbone, respectively.


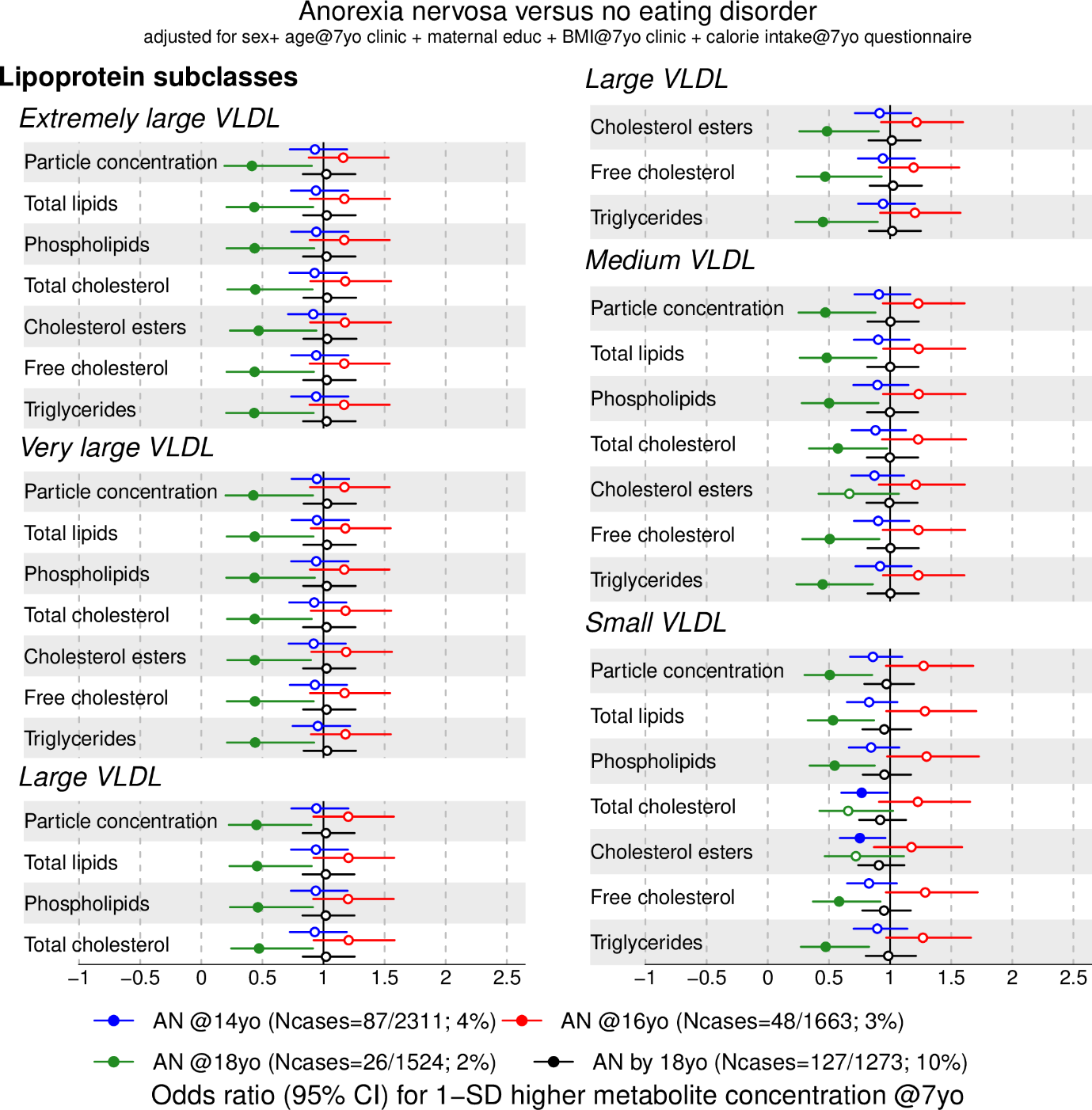


Figure S1b. Estimated odds ratios for anorexia nervosa (AN) at 14, 16, 18 years of age and cumulatively across all three time points according to lipoprotein subclasses particle and lipid concentrations at 7 years. Estimates refer to 1 standard deviation increase in metabolic trait concentration at 7 years. Error bars = 95% confidence intervals (CI). Abbreviations: C=cholesterol; IDL=intermediate-density lipoprotein; LDL=low-density lipoprotein; HDL=high-density lipoprotein; MUFA=monounsaturated fatty acids; PUFA=polyunsaturated fatty acids; VLDL=very-low-density lipoprotein. Note: Filled dot: CI do not include the null. MUFA, PUFA and saturated fatty acid concentrations include all fatty acids detected which have one, more than one, or zero C=C double bonds in their backbone, respectively.


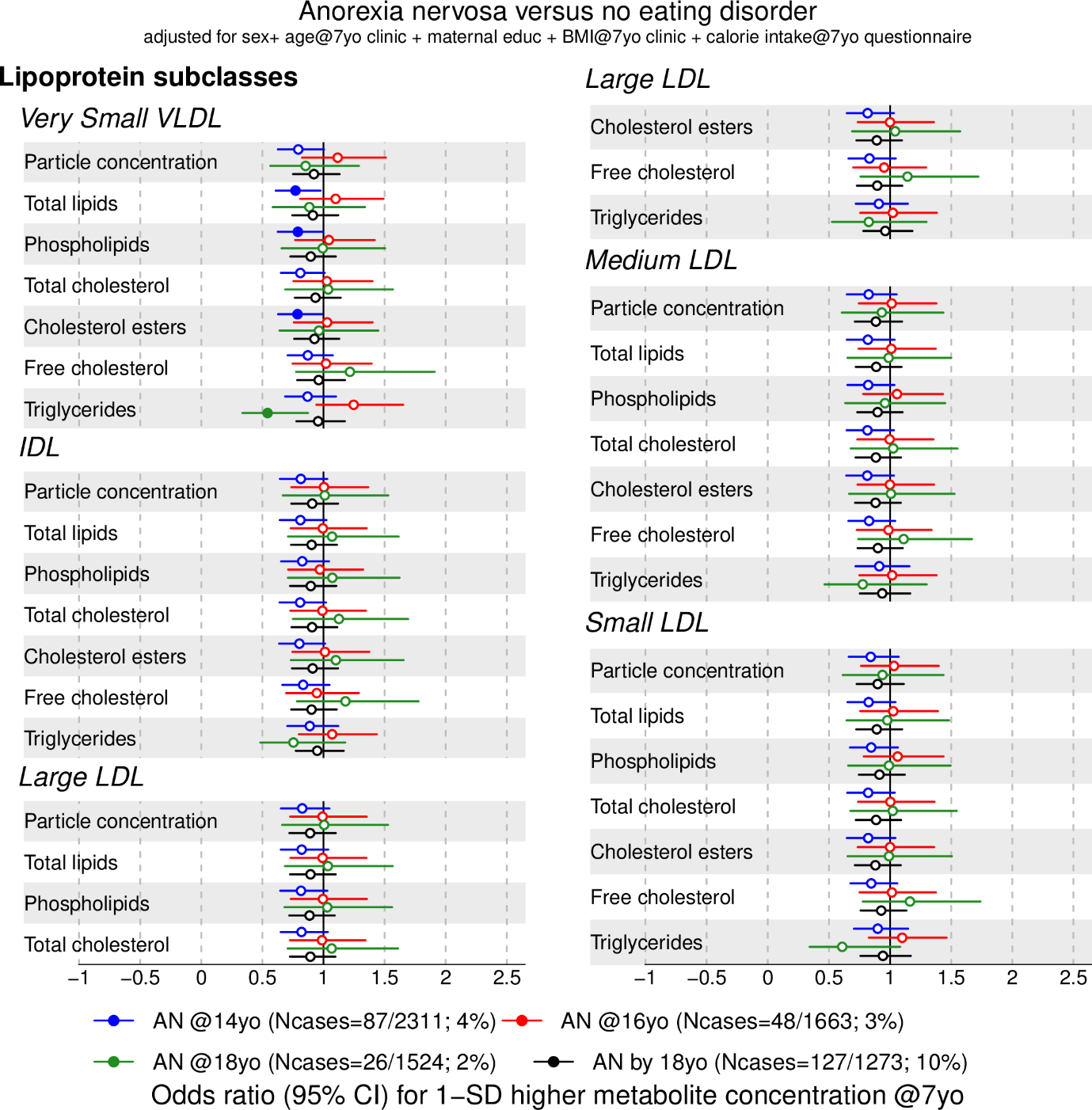


Figure S1c. Estimated odds ratios for anorexia nervosa (AN) at 14, 16, 18 years of age and cumulatively across all three time points according to lipoprotein subclasses particle and lipid concentrations at 7 years. Estimates refer to 1 standard deviation increase in metabolic trait concentration at 7 years. Error bars = 95% confidence intervals (CI). Abbreviations: C=cholesterol; IDL=intermediate-density lipoprotein; LDL=low-density lipoprotein; HDL=high-density lipoprotein; MUFA=monounsaturated fatty acids; PUFA=polyunsaturated fatty acids; VLDL=very-low-density lipoprotein. Note: Filled dot: CI do not include the null. MUFA, PUFA and saturated fatty acid concentrations include all fatty acids detected which have one, more than one, or zero C=C double bonds in their backbone, respectively.


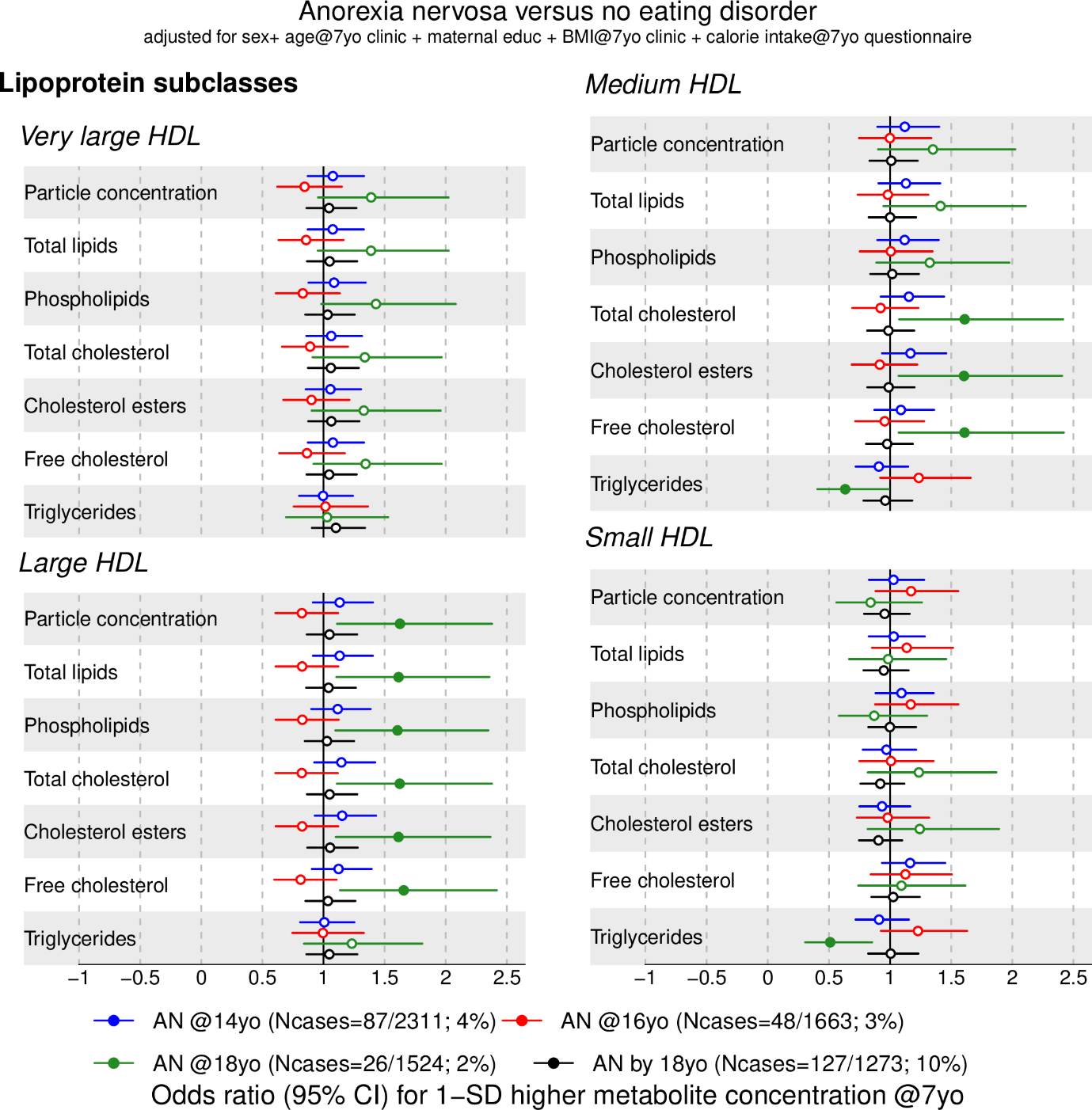


Figure S2a. Estimated odds ratios for anorexia nervosa (AN) at 14 years of age according to metabolic traits concentrations at 7 years. Odds ratio adjusted for: model 1, maternal education and child’s sex and age; model 2, model 1 plus child’s BMI at 7 years; model 3, model 1 plus child’s calorie intake at 7 years; and model 4, model 1 further adjusted for child’s BMI and calorie intake at 7 years. Error bars = 95% confidence intervals (CI). Abbreviations: C=cholesterol; IDL=intermediate-density lipoprotein; LDL=low-density lipoprotein; HDL=high-density lipoprotein; MUFA=monounsaturated fatty acids; PUFA=polyunsaturated fatty acids; VLDL=very-low-density lipoprotein. Note: Filled dot: CI do not include the null. MUFA, PUFA and saturated fatty acid concentrations include all fatty acids detected which have one, more than one, or zero C=C double bonds in their backbone, respectively.

***
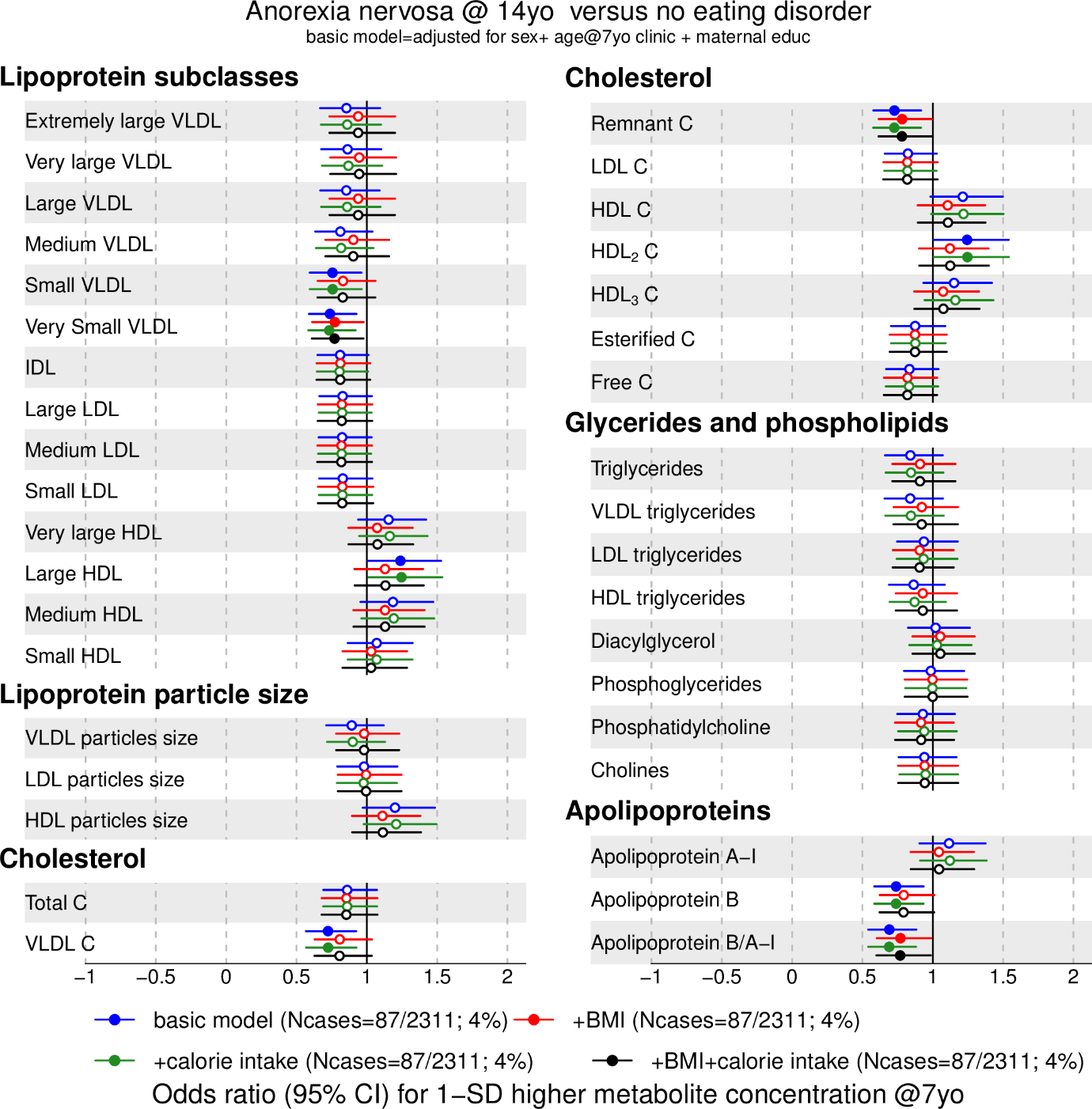
***

Figure S2b. Estimated odds ratios for anorexia nervosa (AN) at 14 years of age according to metabolic traits concentrations at 7 years. Odds ratio adjusted for: model 1, maternal education and child’s sex and age; model 2, model 1 plus child’s BMI at 7 years; model 3, model 1 plus child’s calorie intake at 7 years; and model 4, model 1 further adjusted for child’s BMI and calorie intake at 7 years. Error bars = 95% confidence intervals (CI). Abbreviations: C=cholesterol; IDL=intermediate-density lipoprotein; LDL=low-density lipoprotein; HDL=high-density lipoprotein; MUFA=monounsaturated fatty acids; PUFA=polyunsaturated fatty acids; VLDL=very-low-density lipoprotein. Note: Filled dot: CI do not include the null. MUFA, PUFA and saturated fatty acid concentrations include all fatty acids detected which have one, more than one, or zero C=C double bonds in their backbone, respectively.

***
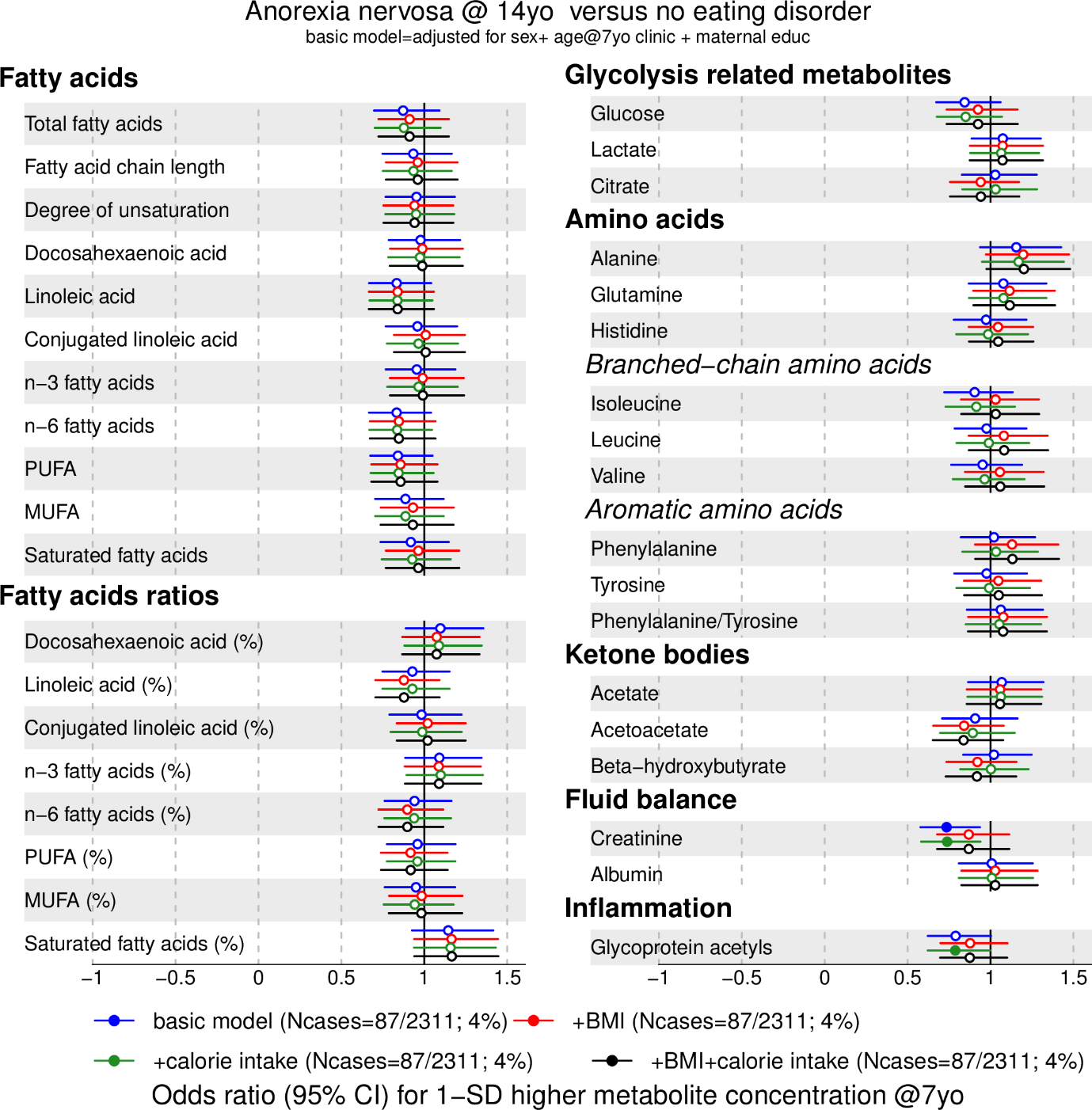
***

Figure S3a. Estimated odds ratios for anorexia nervosa (AN) at 16 years of age according to metabolic traits concentrations at 7 years. Odds ratio adjusted for: model 1, maternal education and child’s sex and age; model 2, model 1 plus child’s BMI at 7 years; model 3, model 1 plus child’s calorie intake at 7 years; and model 4, model 1 further adjusted for child’s BMI and calorie intake at 7 years. Error bars = 95% confidence intervals (CI). Abbreviations: C=cholesterol; IDL=intermediate-density lipoprotein; LDL=low-density lipoprotein; HDL=high-density lipoprotein; MUFA=monounsaturated fatty acids; PUFA=polyunsaturated fatty acids; VLDL=very-low-density lipoprotein. Note: Filled dot: CI do not include the null. MUFA, PUFA and saturated fatty acid concentrations include all fatty acids detected which have one, more than one, or zero C=C double bonds in their backbone, respectively.

***
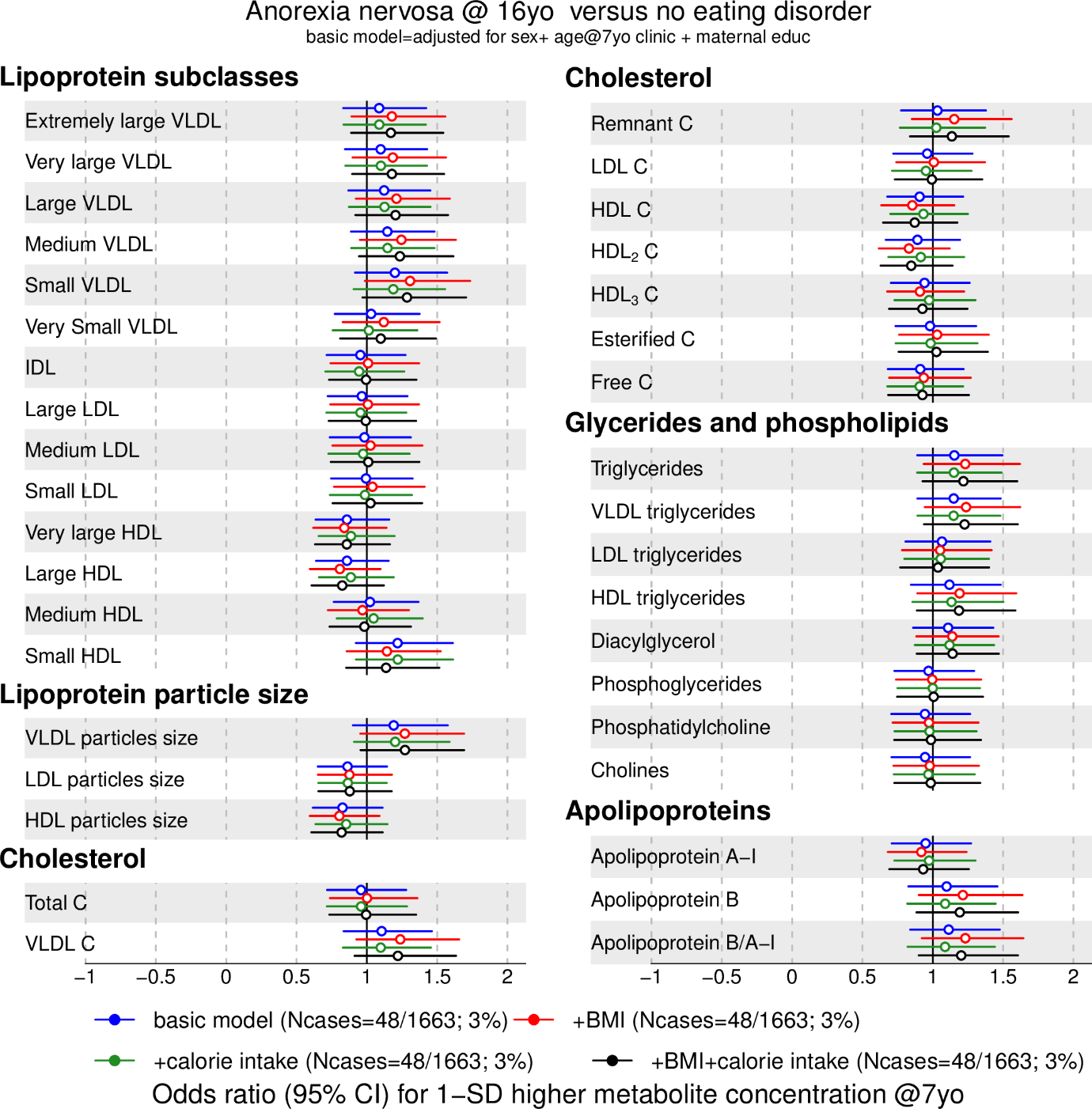
***

Figure S3b. Estimated odds ratios for anorexia nervosa (AN) at 16 years of age according to metabolic traits concentrations at 7 years. Odds ratio adjusted for: model 1, maternal education and child’s sex and age; model 2, model 1 plus child’s BMI at 7 years; model 3, model 1 plus child’s calorie intake at 7 years; and model 4, model 1 further adjusted for child’s BMI and calorie intake at 7 years. Error bars = 95% confidence intervals (CI). Abbreviations: C=cholesterol; IDL=intermediate-density lipoprotein; LDL=low-density lipoprotein; HDL=high-density lipoprotein; MUFA=monounsaturated fatty acids; PUFA=polyunsaturated fatty acids; VLDL=very-low-density lipoprotein. Note: Filled dot: CI do not include the null. MUFA, PUFA and saturated fatty acid concentrations include all fatty acids detected which have one, more than one, or zero C=C double bonds in their backbone, respectively.

***
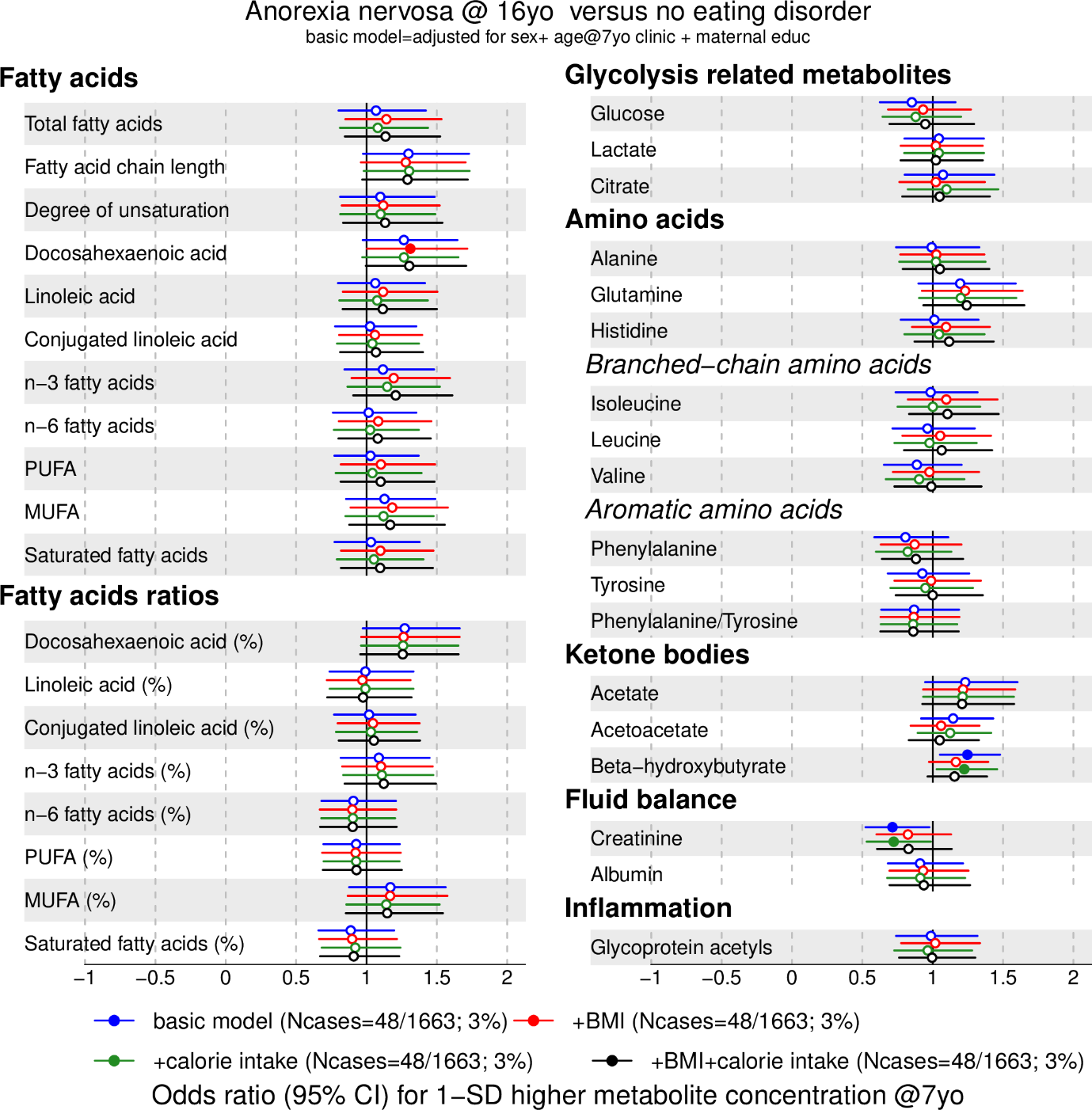
***

Figure S4a. Estimated odds ratios for anorexia nervosa (AN) at 18 years of age according to metabolic traits concentrations at 7 years. Odds ratio adjusted for: model 1, maternal education and child’s sex and age; model 2, model 1 plus child’s BMI at 7 years; model 3, model 1 plus child’s calorie intake at 7 years; and model 4, model 1 further adjusted for child’s BMI and calorie intake at 7 years. Error bars = 95% confidence intervals (CI). Abbreviations: C=cholesterol; IDL=intermediate-density lipoprotein; LDL=low-density lipoprotein; HDL=high-density lipoprotein; MUFA=monounsaturated fatty acids; PUFA=polyunsaturated fatty acids; VLDL=very-low-density lipoprotein. Note: Filled dot: CI do not include the null. MUFA, PUFA and saturated fatty acid concentrations include all fatty acids detected which have one, more than one, or zero C=C double bonds in their backbone, respectively.

***
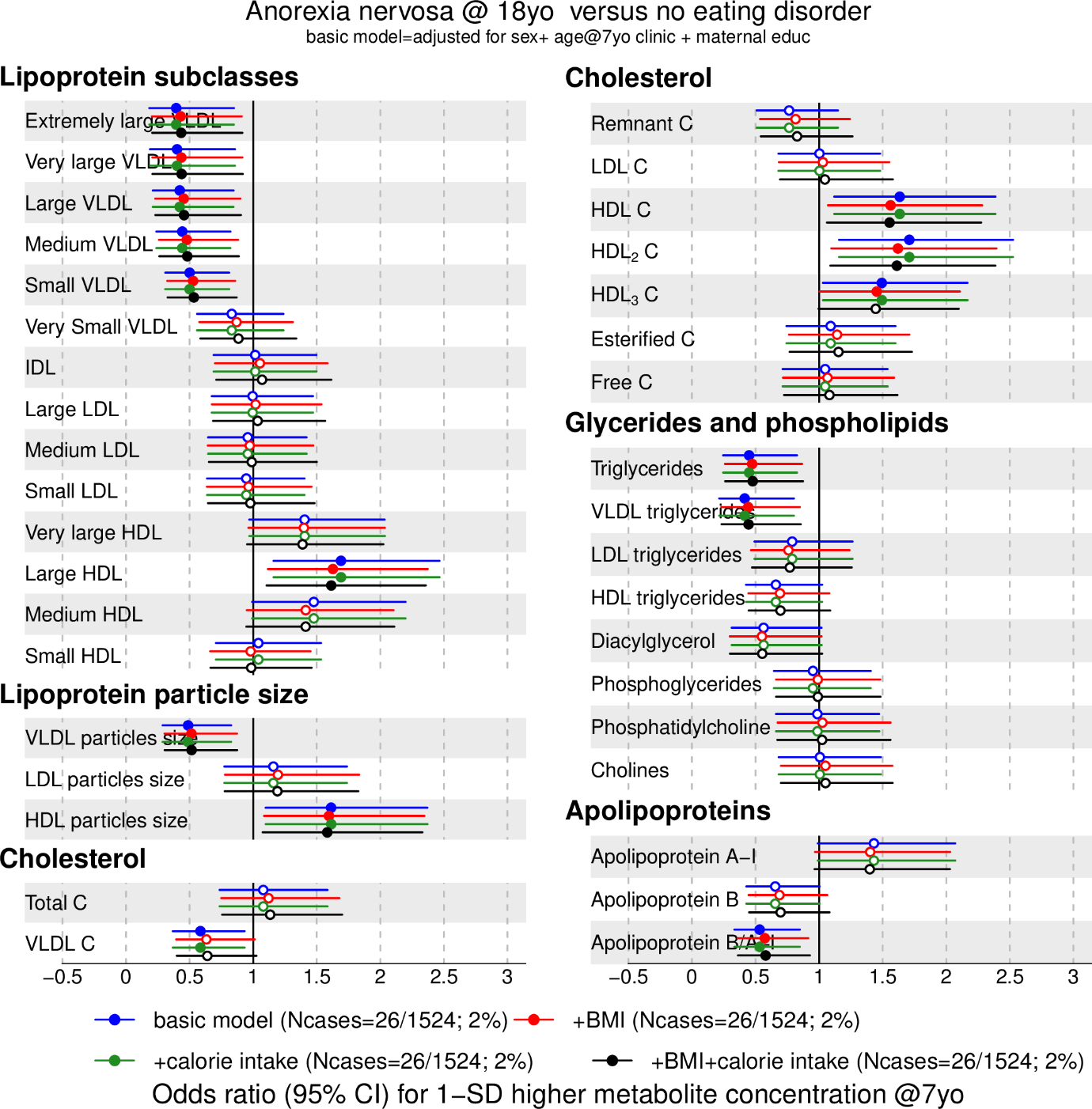
***

Figure S4b. Estimated odds ratios for anorexia nervosa (AN) at 18 years of age according to metabolic traits concentrations at 7 years. Odds ratio adjusted for: model 1, maternal education and child’s sex and age; model 2, model 1 plus child’s BMI at 7 years; model 3, model 1 plus child’s calorie intake at 7 years; and model 4, model 1 further adjusted for child’s BMI and calorie intake at 7 years. Error bars = 95% confidence intervals (CI). Abbreviations: C=cholesterol; IDL=intermediate-density lipoprotein; LDL=low-density lipoprotein; HDL=high-density lipoprotein; MUFA=monounsaturated fatty acids; PUFA=polyunsaturated fatty acids; VLDL=very-low-density lipoprotein. Note: Filled dot: CI do not include the null. MUFA, PUFA and saturated fatty acid concentrations include all fatty acids detected which have one, more than one, or zero C=C double bonds in their backbone, respectively.


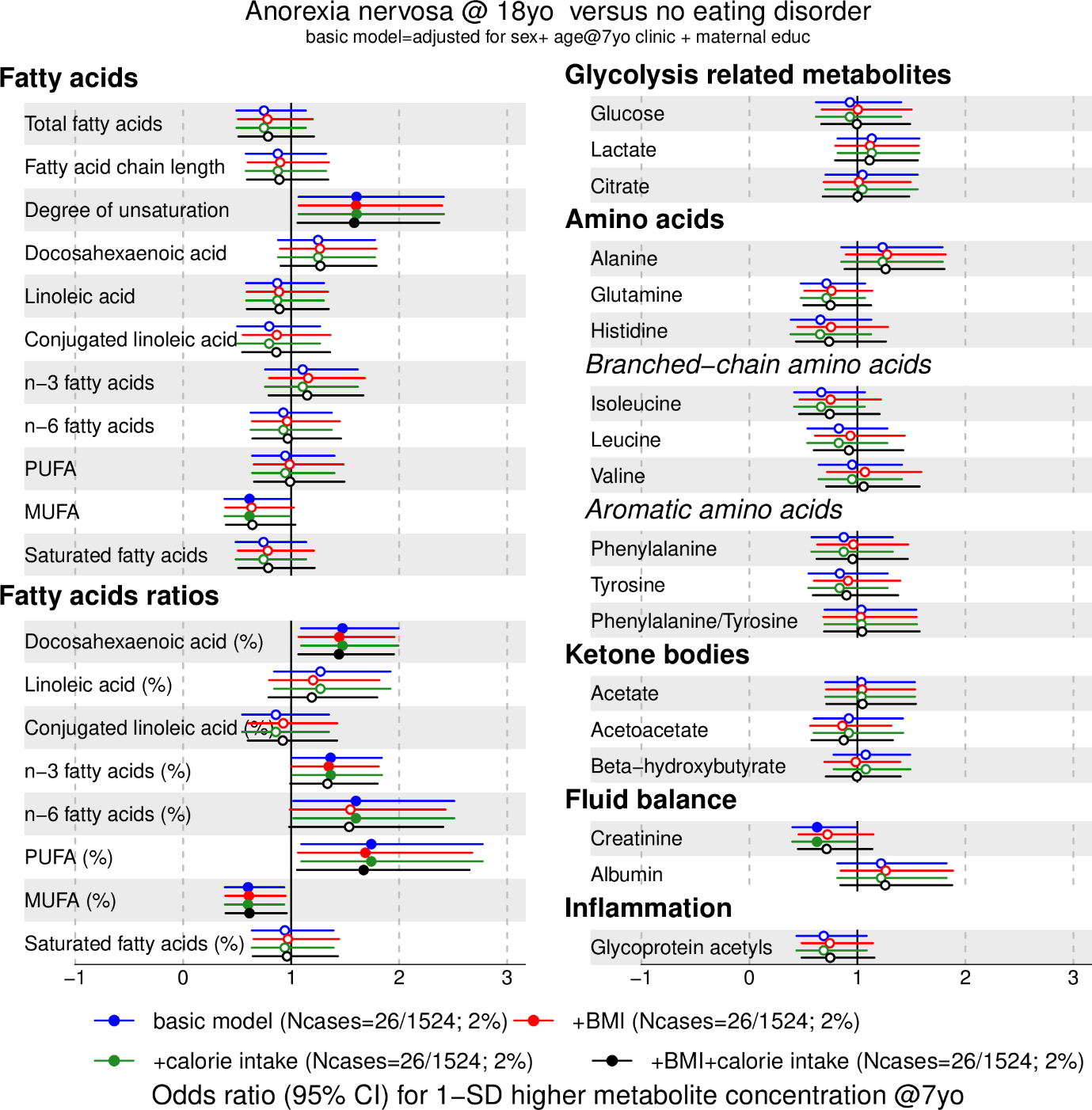


Figure S5a. Estimated odds ratios for anorexia nervosa (AN) by 18 years of age (i.e. cumulatively across 14, 16, 18 years old) according to metabolic traits concentrations at 7 years. Odds ratio adjusted for: model 1, maternal education and child’s sex and age; model 2, model 1 plus child’s BMI at 7 years; model 3, model 1 plus child’s calorie intake at 7 years; and model 4, model 1 further adjusted for child’s BMI and calorie intake at 7 years. Error bars = 95% confidence intervals (CI). Abbreviations: C=cholesterol; IDL=intermediate-density lipoprotein; LDL=low-density lipoprotein; HDL=high-density lipoprotein; MUFA=monounsaturated fatty acids; PUFA=polyunsaturated fatty acids; VLDL=very-low-density lipoprotein. Note: Filled dot: CI do not include the null. MUFA, PUFA and saturated fatty acid concentrations include all fatty acids detected which have one, more than one, or zero C=C double bonds in their backbone, respectively.

***
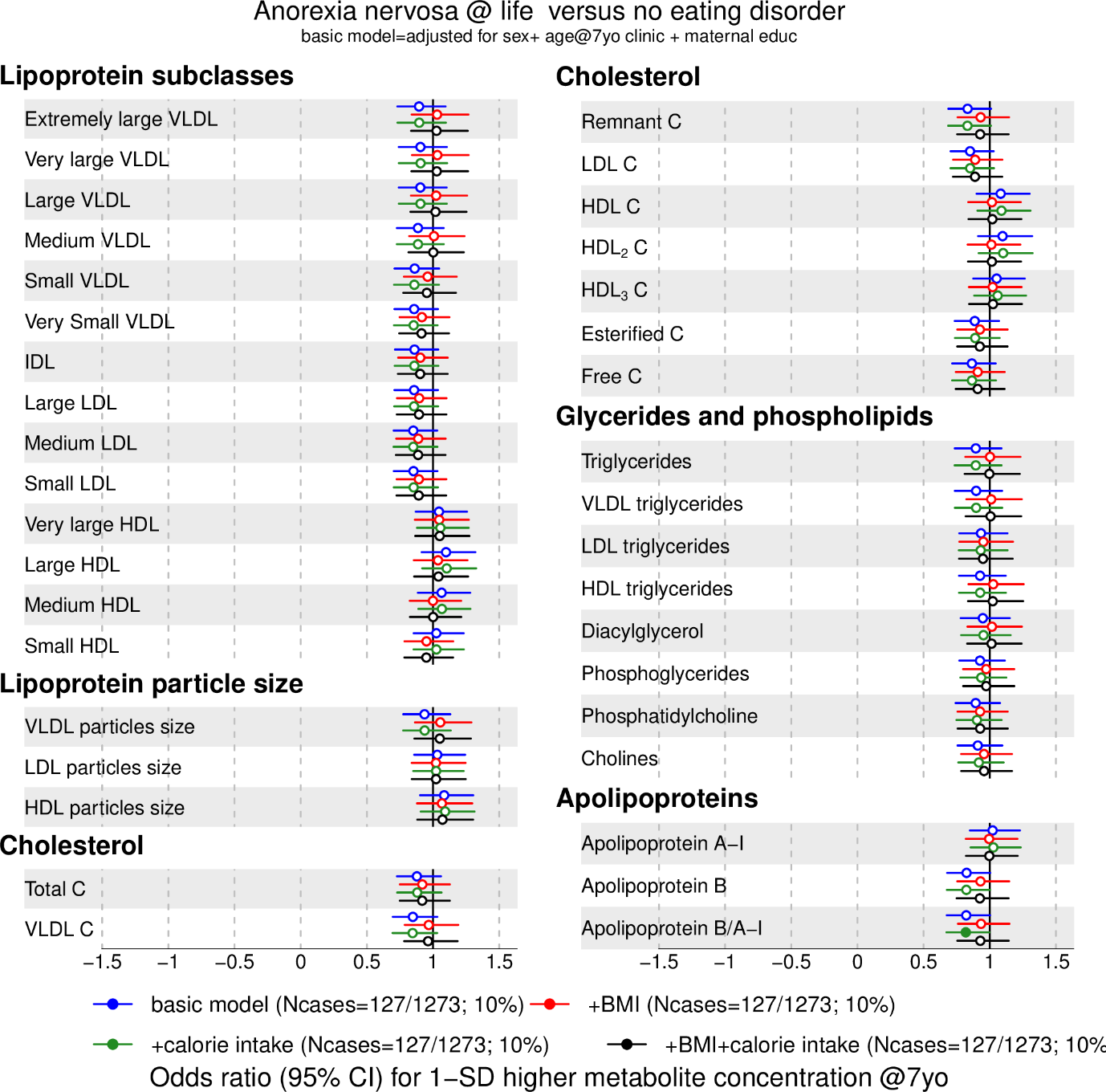
***

Figure S5b. Estimated odds ratios for anorexia nervosa (AN) by 18 years of age (i.e. cumulatively across 14, 16, 18 years old) according to metabolic traits concentrations at 7 years. Odds ratio adjusted for: model 1, maternal education and child’s sex and age; model 2, model 1 plus child’s BMI at 7 years; model 3, model 1 plus child’s calorie intake at 7 years; and model 4, model 1 further adjusted for child’s BMI and calorie intake at 7 years. Error bars = 95% confidence intervals (CI). Abbreviations: C=cholesterol; IDL=intermediate-density lipoprotein; LDL=low-density lipoprotein; HDL=high-density lipoprotein; MUFA=monounsaturated fatty acids; PUFA=polyunsaturated fatty acids; VLDL=very-low-density lipoprotein. Note: Filled dot: CI do not include the null. MUFA, PUFA and saturated fatty acid concentrations include all fatty acids detected which have one, more than one, or zero C=C double bonds in their backbone, respectively.

***
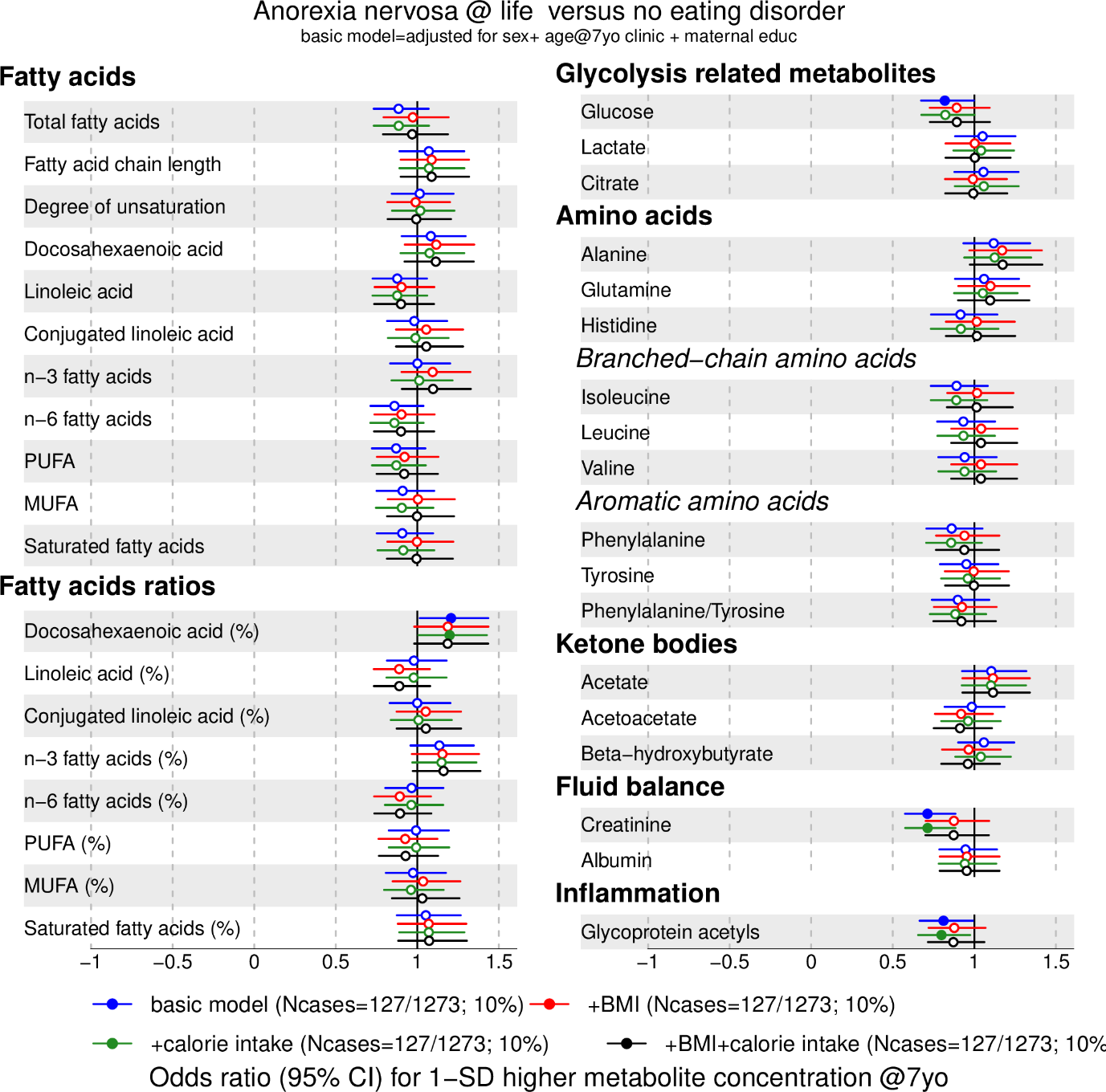
***

Figure S6a. Estimated odds ratios for binge-eating disorder (BED) at 14, 16, 18 years of age and cumulatively across all three time points according to lipoprotein subclasses particle and lipid concentrations at 7 years. Estimates refer to 1 standard deviation increase in metabolic trait concentration at 7 years. Error bars = 95% confidence intervals (CI). Abbreviations: C=cholesterol; IDL=intermediate-density lipoprotein; LDL=low-density lipoprotein; HDL=high-density lipoprotein; MUFA=monounsaturated fatty acids; PUFA=polyunsaturated fatty acids; VLDL=very-low-density lipoprotein. Note: Filled dot: CI do not include the null. MUFA, PUFA and saturated fatty acid concentrations include all fatty acids detected which have one, more than one, or zero C=C double bonds in their backbone, respectively.


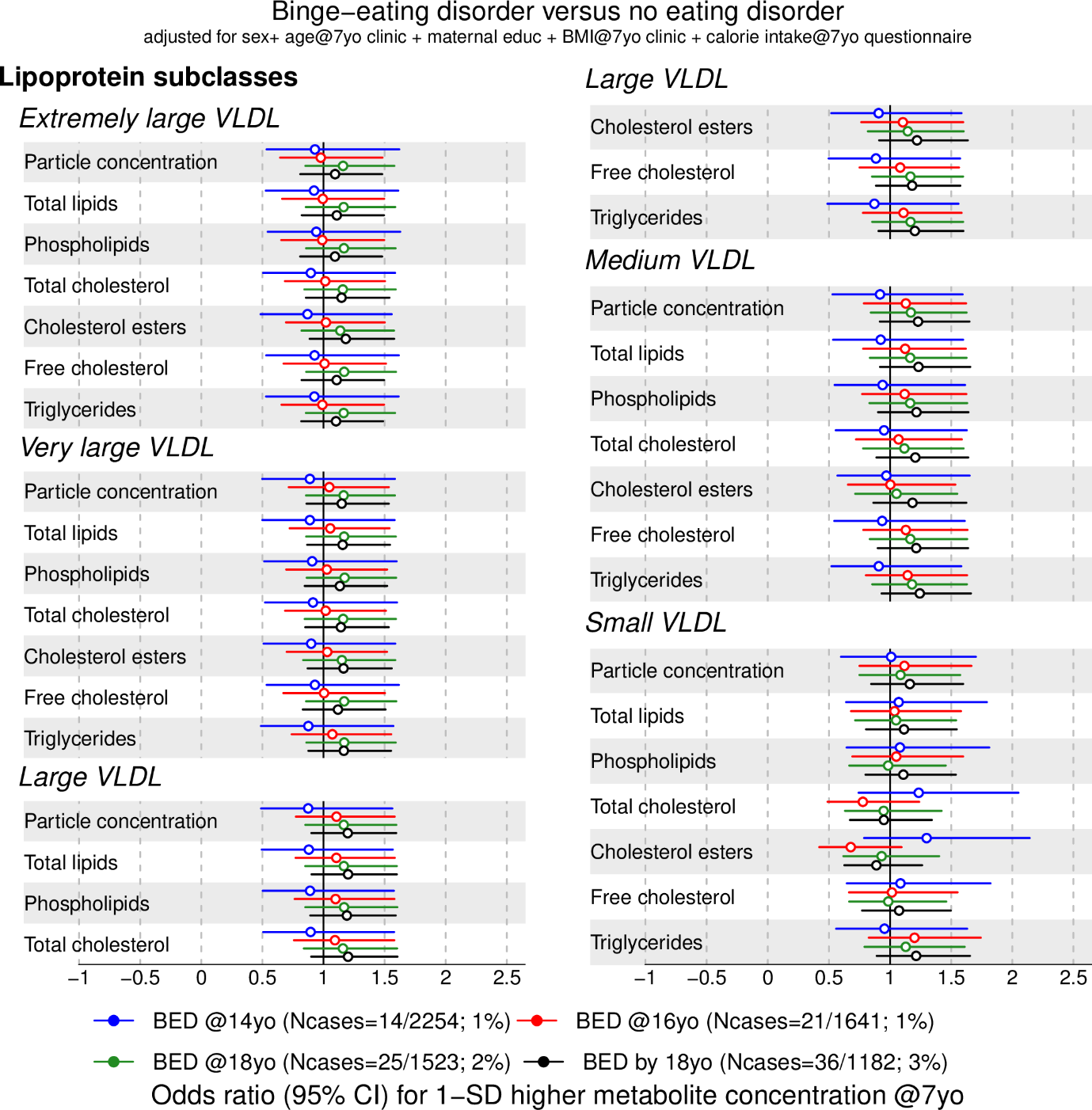


Figure S6b. Estimated odds ratios for binge-eating disorder (BED) at 14, 16, 18 years of age and cumulatively across all three time points according to lipoprotein subclasses particle and lipid concentrations at 7 years. Estimates refer to 1 standard deviation increase in metabolic trait concentration at 7 years. Error bars = 95% confidence intervals (CI). Abbreviations: C=cholesterol; IDL=intermediate-density lipoprotein; LDL=low-density lipoprotein; HDL=high-density lipoprotein; MUFA=monounsaturated fatty acids; PUFA=polyunsaturated fatty acids; VLDL=very-low-density lipoprotein. Note: Filled dot: CI do not include the null. MUFA, PUFA and saturated fatty acid concentrations include all fatty acids detected which have one, more than one, or zero C=C double bonds in their backbone, respectively.


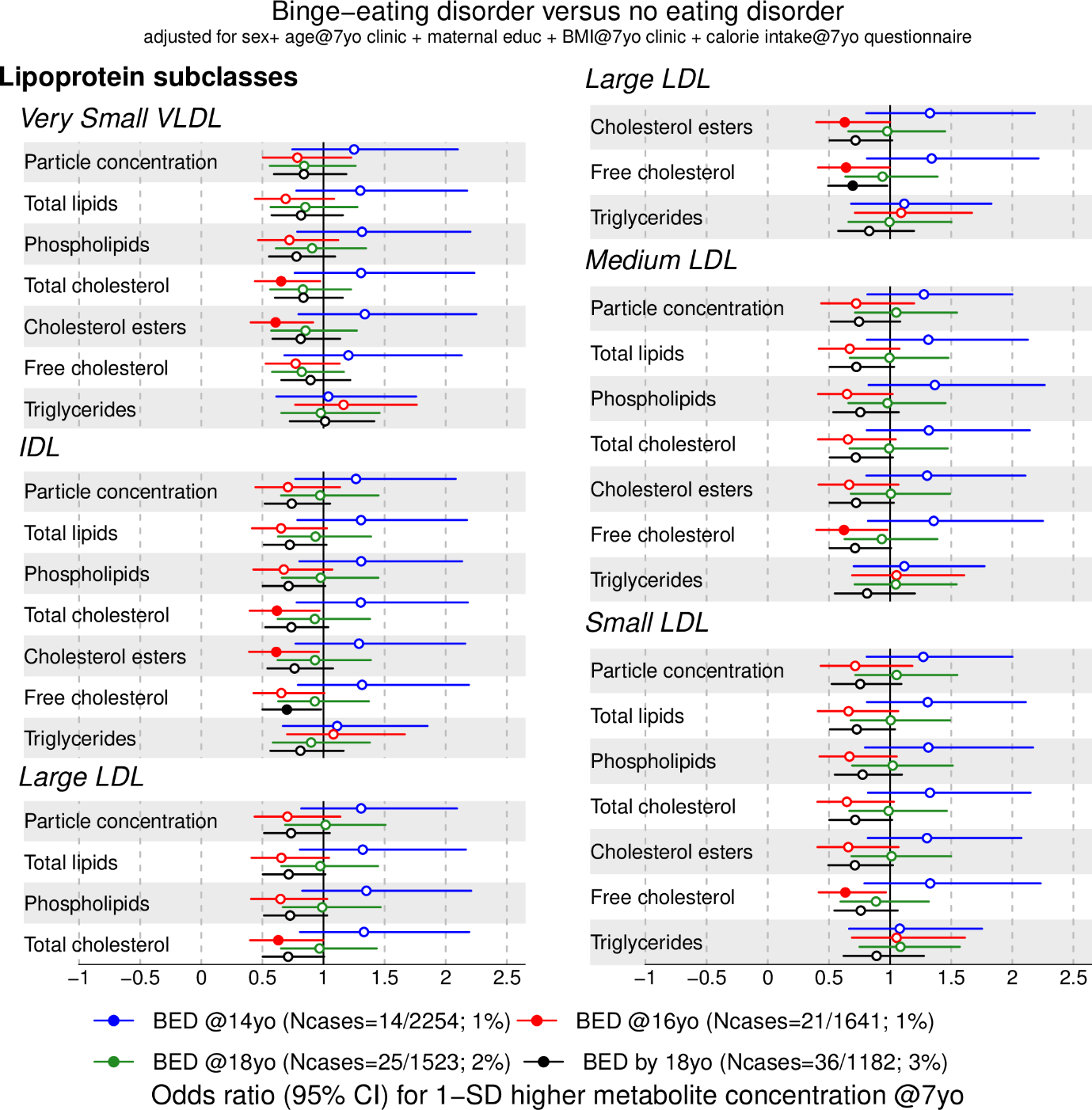


Figure S6c. Estimated odds ratios for binge-eating disorder (BED) at 14, 16, 18 years of age and cumulatively across all three time points according to lipoprotein subclasses particle and lipid concentrations at 7 years. Estimates refer to 1 standard deviation increase in metabolic trait concentration at 7 years. Error bars = 95% confidence intervals (CI). Abbreviations: C=cholesterol; IDL=intermediate-density lipoprotein; LDL=low-density lipoprotein; HDL=high-density lipoprotein; MUFA=monounsaturated fatty acids; PUFA=polyunsaturated fatty acids; VLDL=very-low-density lipoprotein. Note: Filled dot: CI do not include the null. MUFA, PUFA and saturated fatty acid concentrations include all fatty acids detected which have one, more than one, or zero C=C double bonds in their backbone, respectively.


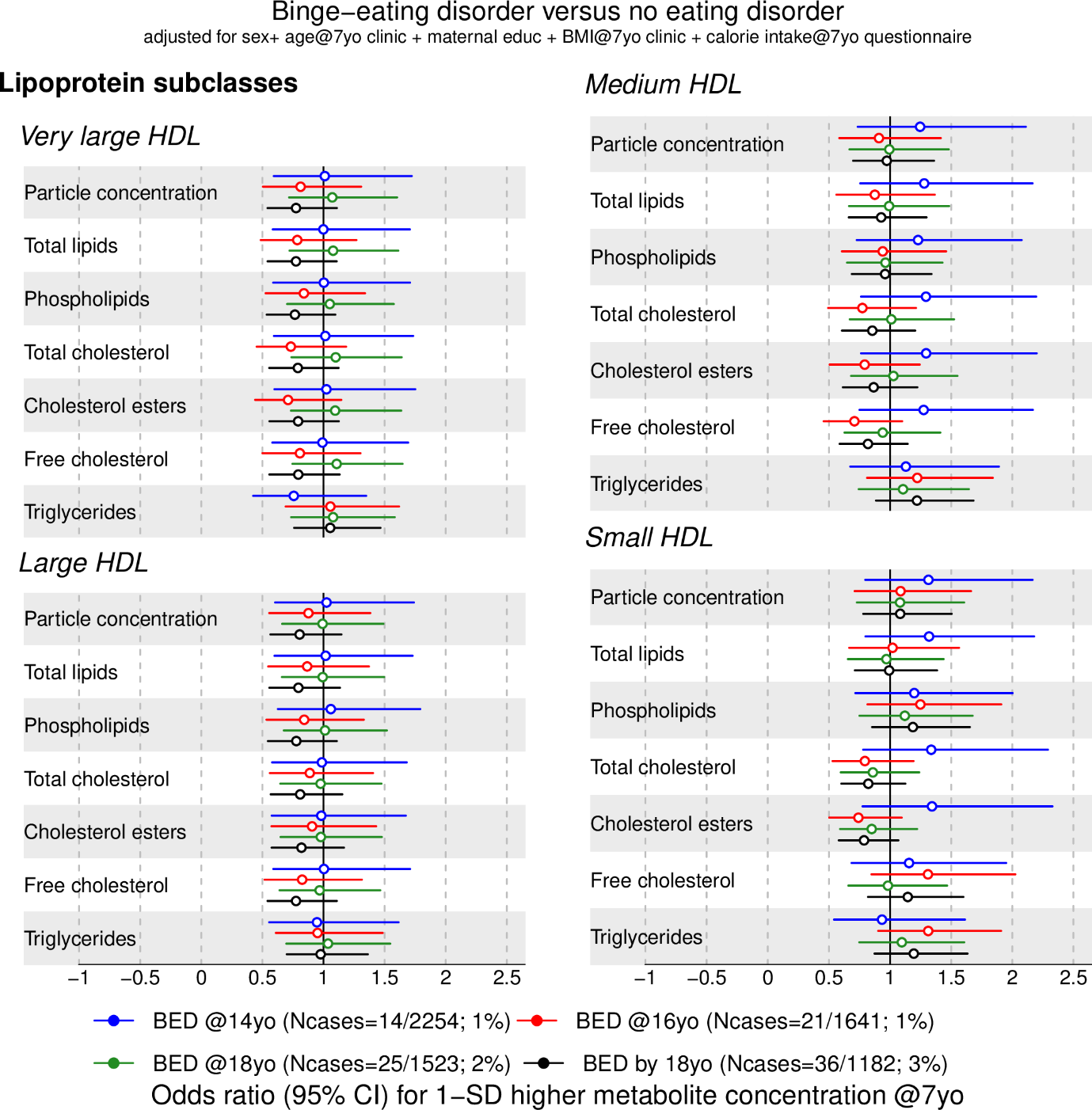


Figure S7a. Estimated odds ratios for binge-eating disorder (BED) at 14 years of age according to metabolic traits concentrations at 7 years. Odds ratio adjusted for: model 1, maternal education and child’s sex and age; model 2, model 1 plus child’s BMI at 7 years; model 3, model 1 plus child’s calorie intake at 7 years; and model 4, model 1 further adjusted for child’s BMI and calorie intake at 7 years. Error bars = 95% confidence intervals (CI). Abbreviations: C=cholesterol; IDL=intermediate-density lipoprotein; LDL=low-density lipoprotein; HDL=high-density lipoprotein; MUFA=monounsaturated fatty acids; PUFA=polyunsaturated fatty acids; VLDL=very-low-density lipoprotein. Note: Filled dot: CI do not include the null. MUFA, PUFA and saturated fatty acid concentrations include all fatty acids detected which have one, more than one, or zero C=C double bonds in their backbone, respectively.

***
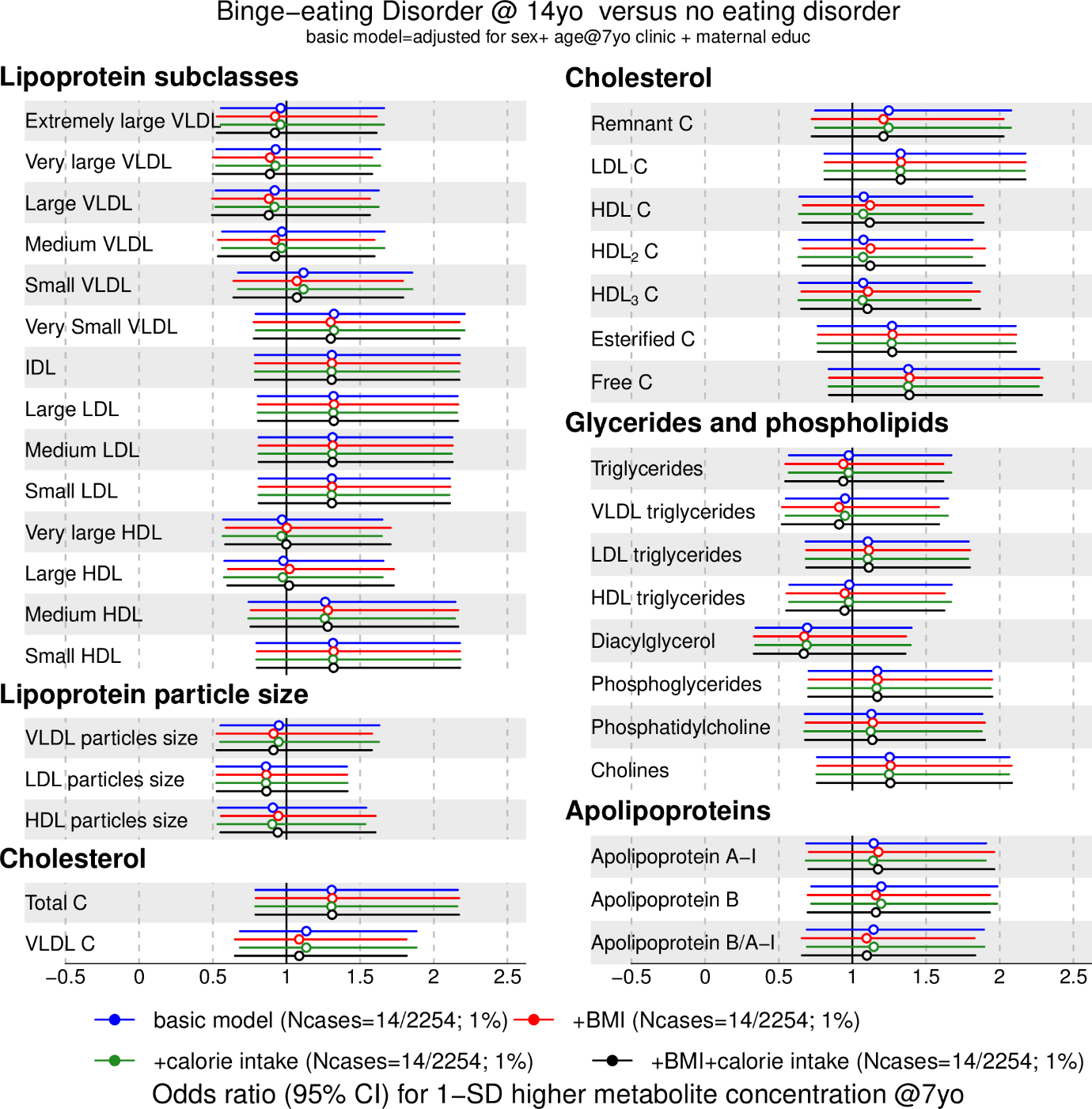
***

Figure S7b. Estimated odds ratios for binge-eating disorder (BED) at 14 years of age according to metabolic traits concentrations at 7 years. Odds ratio adjusted for: model 1, maternal education and child’s sex and age; model 2, model 1 plus child’s BMI at 7 years; model 3, model 1 plus child’s calorie intake at 7 years; and model 4, model 1 further adjusted for child’s BMI and calorie intake at 7 years. Error bars = 95% confidence intervals (CI). Abbreviations: C=cholesterol; IDL=intermediate-density lipoprotein; LDL=low-density lipoprotein; HDL=high-density lipoprotein; MUFA=monounsaturated fatty acids; PUFA=polyunsaturated fatty acids; VLDL=very-low-density lipoprotein. Note: Filled dot: CI do not include the null. MUFA, PUFA and saturated fatty acid concentrations include all fatty acids detected which have one, more than one, or zero C=C double bonds in their backbone, respectively.

*
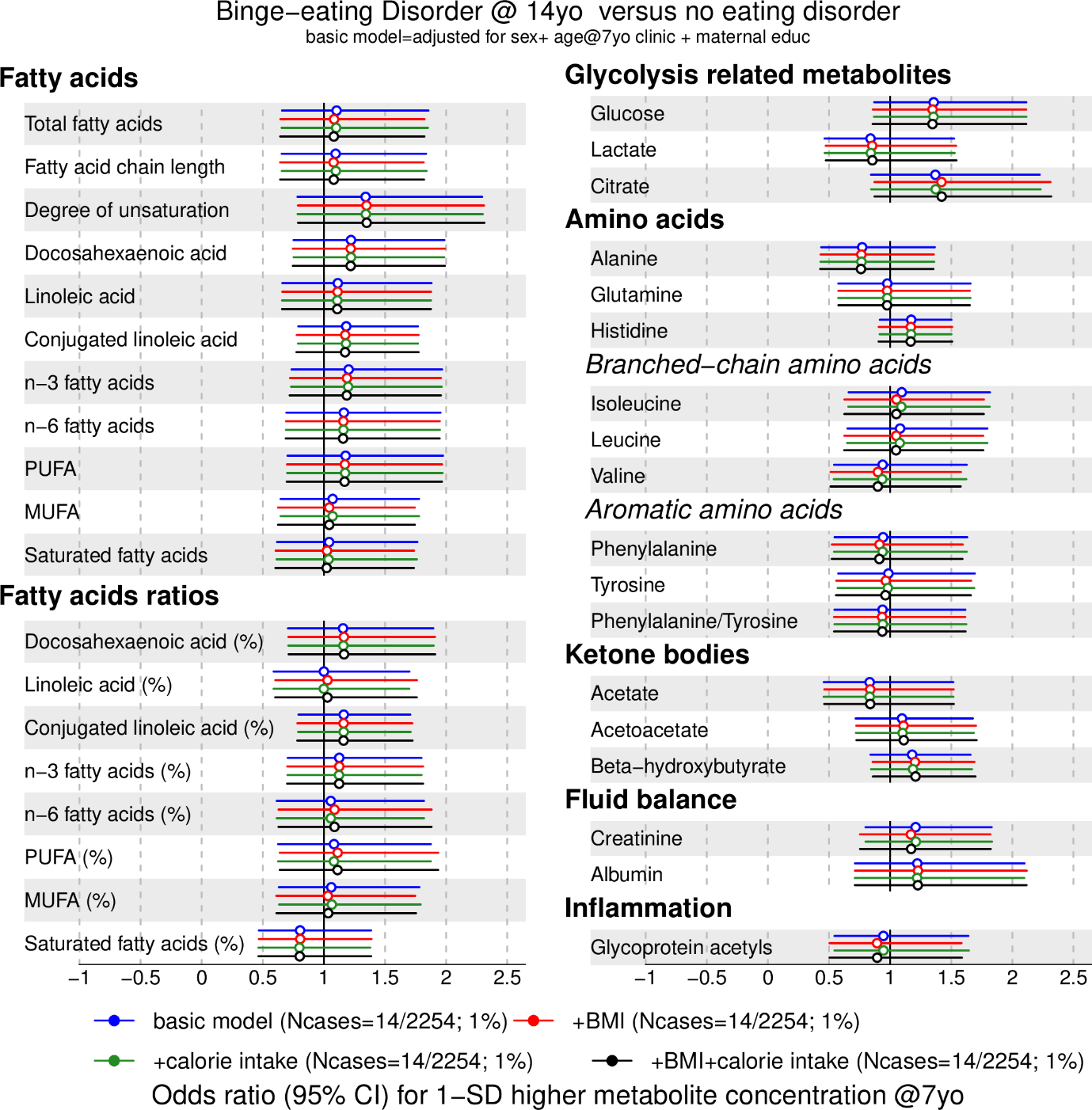
*

Figure S8a. Estimated odds ratios for binge-eating disorder (BED) at 16 years of age according to metabolic traits concentrations at 7 years. Odds ratio adjusted for: model 1, maternal education and child’s sex and age; model 2, model 1 plus child’s BMI at 7 years; model 3, model 1 plus child’s calorie intake at 7 years; and model 4, model 1 further adjusted for child’s BMI and calorie intake at 7 years. Error bars = 95% confidence intervals (CI). Abbreviations: C=cholesterol; IDL=intermediate-density lipoprotein; LDL=low-density lipoprotein; HDL=high-density lipoprotein; MUFA=monounsaturated fatty acids; PUFA=polyunsaturated fatty acids; VLDL=very-low-density lipoprotein. Note: Filled dot: CI do not include the null. MUFA, PUFA and saturated fatty acid concentrations include all fatty acids detected which have one, more than one, or zero C=C double bonds in their backbone, respectively.


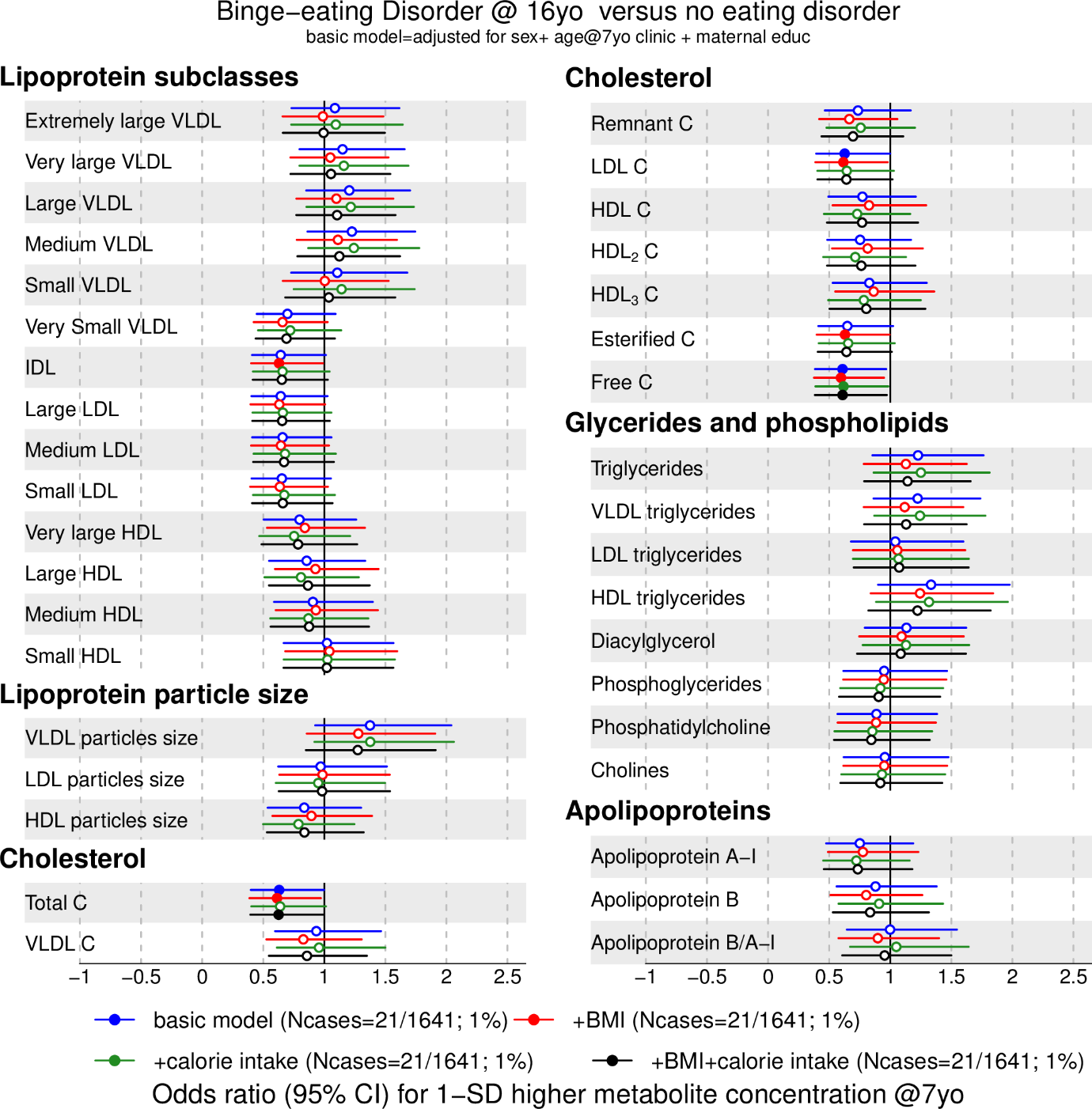


Figure S8b. Estimated odds ratios for binge-eating disorder (BED) at 16 years of age according to metabolic traits concentrations at 7 years. Odds ratio adjusted for: model 1, maternal education and child’s sex and age; model 2, model 1 plus child’s BMI at 7 years; model 3, model 1 plus child’s calorie intake at 7 years; and model 4, model 1 further adjusted for child’s BMI and calorie intake at 7 years. Error bars = 95% confidence intervals (CI). Abbreviations: C=cholesterol; IDL=intermediate-density lipoprotein; LDL=low-density lipoprotein; HDL=high-density lipoprotein; MUFA=monounsaturated fatty acids; PUFA=polyunsaturated fatty acids; VLDL=very-low-density lipoprotein. Note: Filled dot: CI do not include the null. MUFA, PUFA and saturated fatty acid concentrations include all fatty acids detected which have one, more than one, or zero C=C double bonds in their backbone, respectively.

*
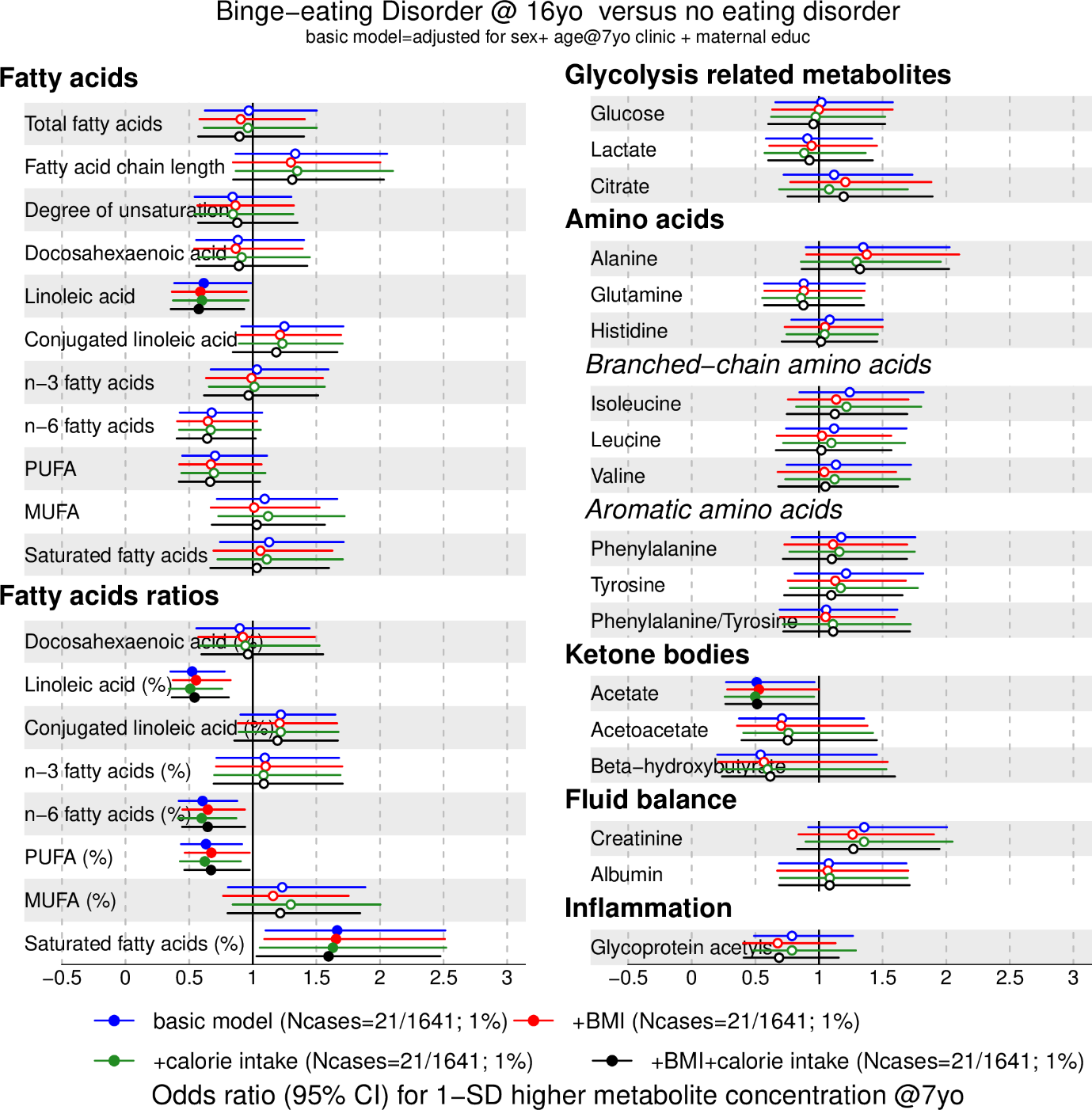
*

Figure S9a. Estimated odds ratios for binge-eating disorder (BED) at 18 years of age according to metabolic traits concentrations at 7 years. Odds ratio adjusted for: model 1, maternal education and child’s sex and age; model 2, model 1 plus child’s BMI at 7 years; model 3, model 1 plus child’s calorie intake at 7 years; and model 4, model 1 further adjusted for child’s BMI and calorie intake at 7 years. Error bars = 95% confidence intervals (CI). Abbreviations: C=cholesterol; IDL=intermediate-density lipoprotein; LDL=low-density lipoprotein; HDL=high-density lipoprotein; MUFA=monounsaturated fatty acids; PUFA=polyunsaturated fatty acids; VLDL=very-low-density lipoprotein. Note: Filled dot: CI do not include the null. MUFA, PUFA and saturated fatty acid concentrations include all fatty acids detected which have one, more than one, or zero C=C double bonds in their backbone, respectively.

***
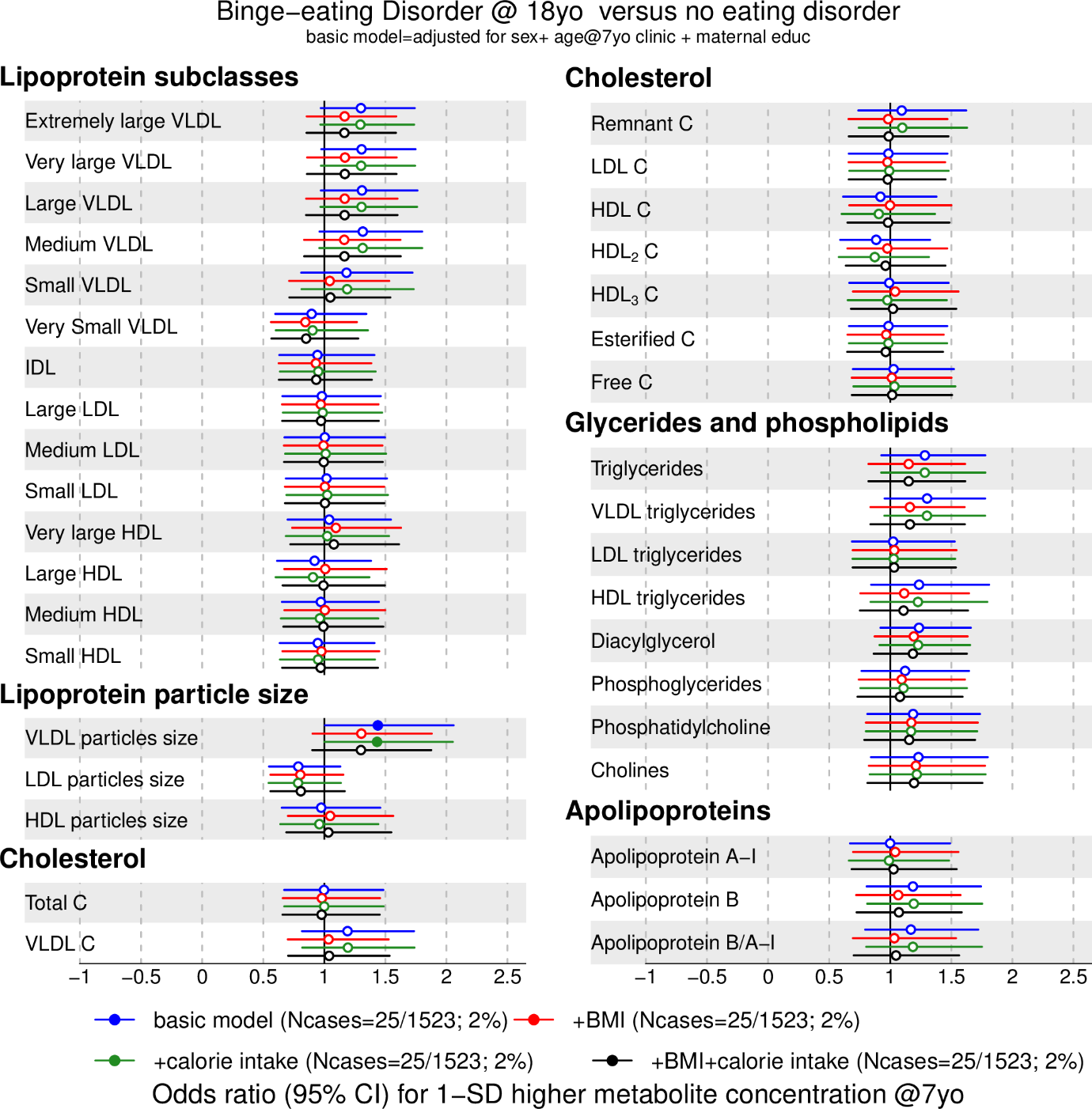
***

Figure S9b. Estimated odds ratios for binge-eating disorder (BED) at 18 years of age according to metabolic traits concentrations at 7 years. Odds ratio adjusted for: model 1, maternal education and child’s sex and age; model 2, model 1 plus child’s BMI at 7 years; model 3, model 1 plus child’s calorie intake at 7 years; and model 4, model 1 further adjusted for child’s BMI and calorie intake at 7 years. Error bars = 95% confidence intervals (CI). Abbreviations: C=cholesterol; IDL=intermediate-density lipoprotein; LDL=low-density lipoprotein; HDL=high-density lipoprotein; MUFA=monounsaturated fatty acids; PUFA=polyunsaturated fatty acids; VLDL=very-low-density lipoprotein. Note: Filled dot: CI do not include the null. MUFA, PUFA and saturated fatty acid concentrations include all fatty acids detected which have one, more than one, or zero C=C double bonds in their backbone, respectively.

*
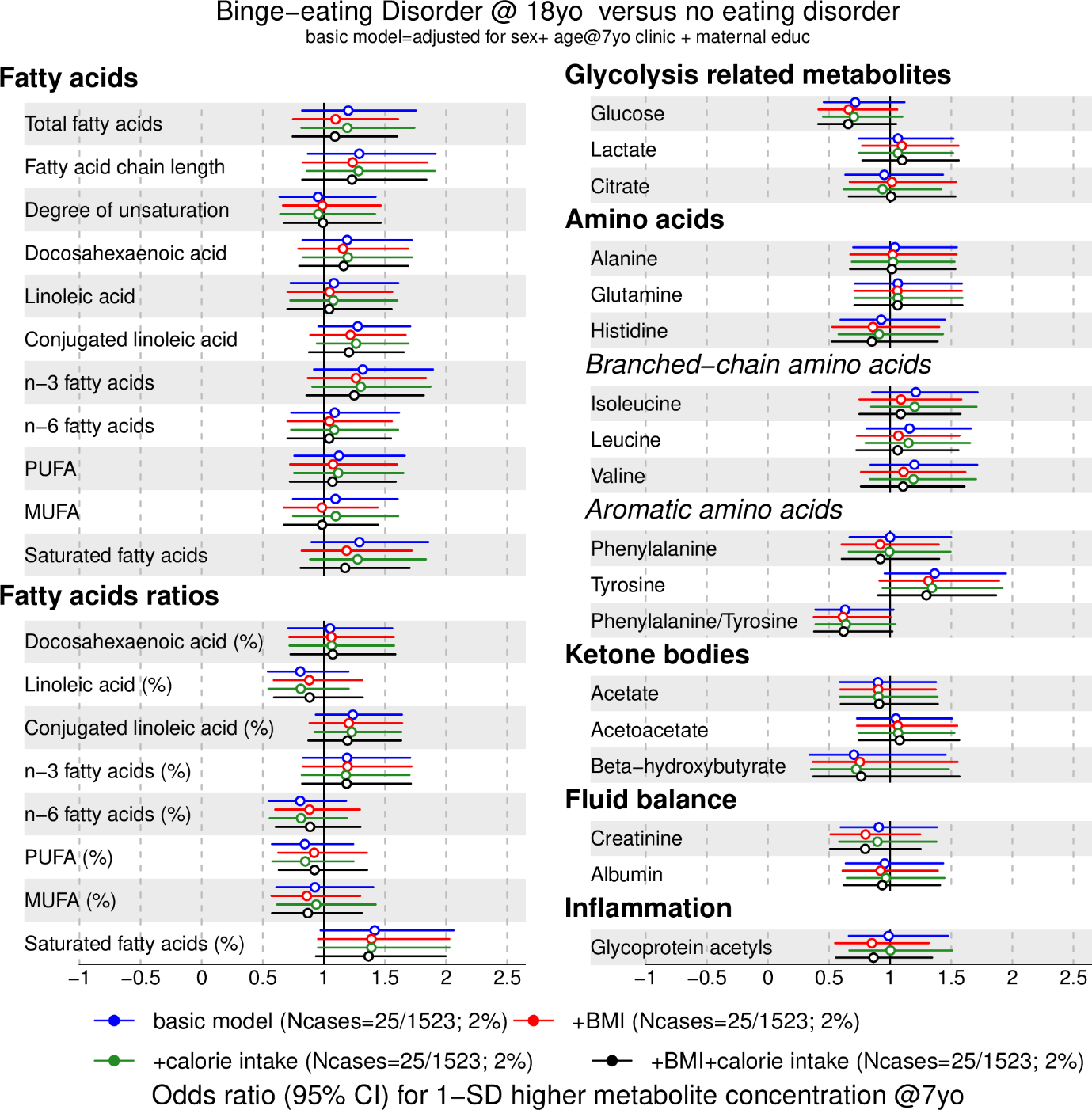
*

Figure S10a. Estimated odds ratios for binge-eating disorder (BED) by 18 years of age (i.e. cumulatively across 14, 16, 18 years old) according to metabolic traits concentrations at 7 years. Odds ratio adjusted for: model 1, maternal education and child’s sex and age; model 2, model 1 plus child’s BMI at 7 years; model 3, model 1 plus child’s calorie intake at 7 years; and model 4, model 1 further adjusted for child’s BMI and calorie intake at 7 years. Error bars = 95% confidence intervals (CI). Abbreviations: C=cholesterol; IDL=intermediate-density lipoprotein; LDL=low-density lipoprotein; HDL=high-density lipoprotein; MUFA=monounsaturated fatty acids; PUFA=polyunsaturated fatty acids; VLDL=very-low-density lipoprotein. Note: Filled dot: CI do not include the null. MUFA, PUFA and saturated fatty acid concentrations include all fatty acids detected which have one, more than one, or zero C=C double bonds in their backbone, respectively.

***
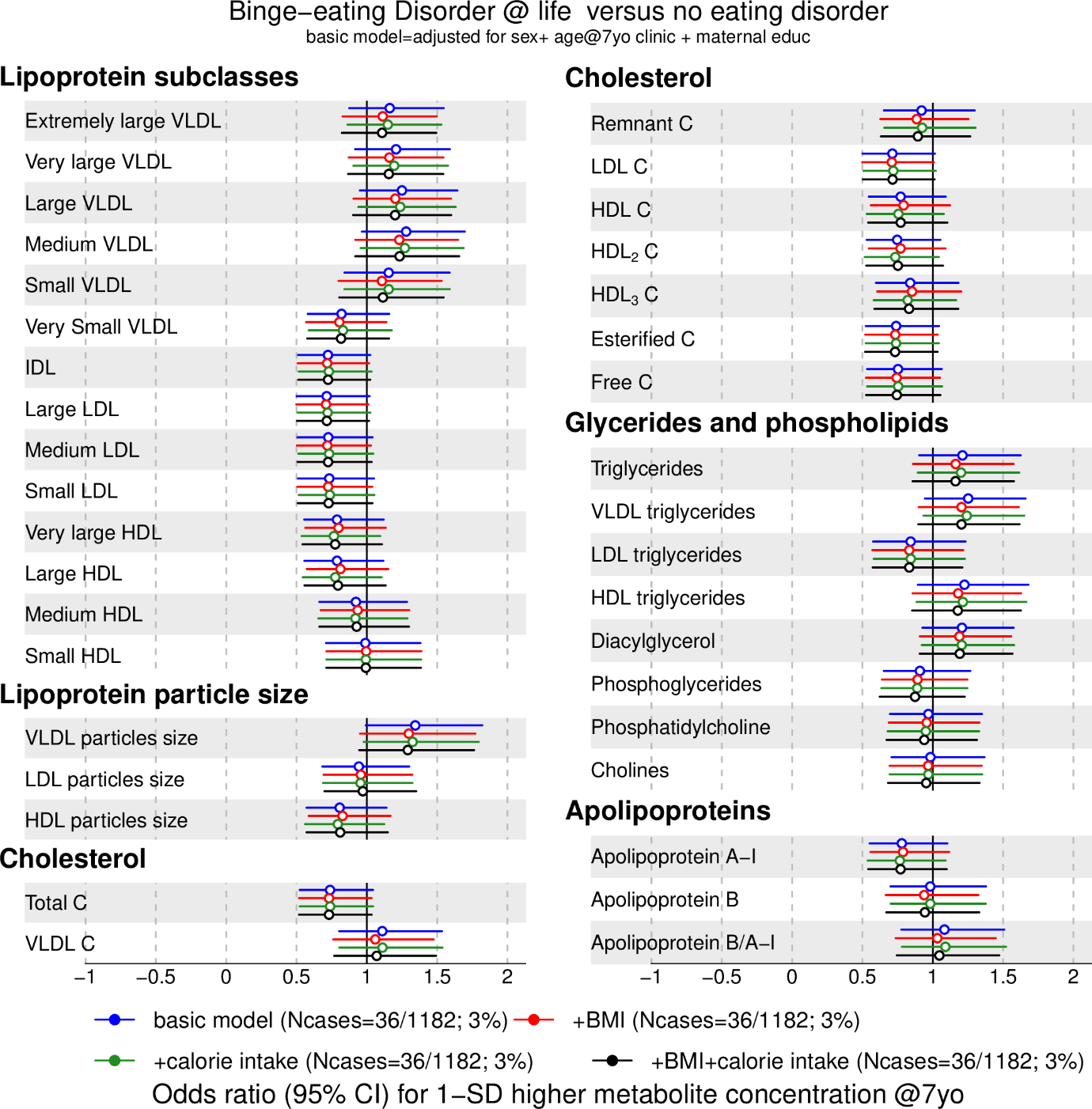
***

Figure S10b. Estimated odds ratios for binge-eating disorder (BED) by 18 years of age (i.e. cumulatively across 14, 16, 18 years old) according to metabolic traits concentrations at 7 years. Odds ratio adjusted for: model 1, maternal education and child’s sex and age; model 2, model 1 plus child’s BMI at 7 years; model 3, model 1 plus child’s calorie intake at 7 years; and model 4, model 1 further adjusted for child’s BMI and calorie intake at 7 years. Error bars = 95% confidence intervals (CI). Abbreviations: C=cholesterol; IDL=intermediate-density lipoprotein; LDL=low-density lipoprotein; HDL=high-density lipoprotein; MUFA=monounsaturated fatty acids; PUFA=polyunsaturated fatty acids; VLDL=very-low-density lipoprotein. Note: Filled dot: CI do not include the null. MUFA, PUFA and saturated fatty acid concentrations include all fatty acids detected which have one, more than one, or zero C=C double bonds in their backbone, respectively.

***
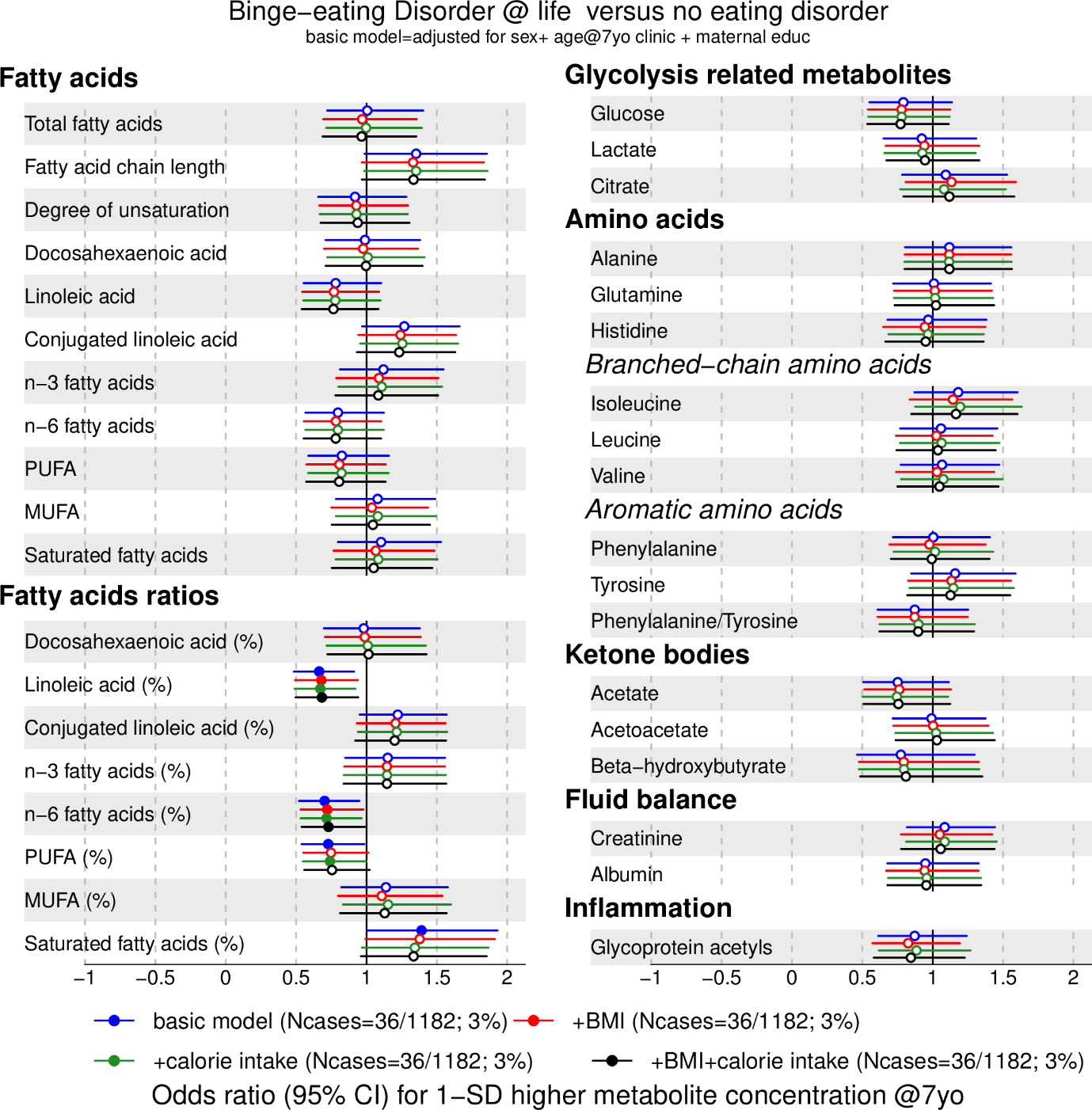
***

Figure S11a. Estimated odds ratios for binge-eating disorder (BED) and anorexia nervosa (AN) at 14, 16, 18 years of age and cumulatively across all three time points according to metabolic traits concentration at 7 years. Estimates refer to 1 standard deviation increase in metabolic trait concentration at 7 years. Error bars = 95% confidence intervals (CI). Abbreviations: C=cholesterol; IDL=intermediate-density lipoprotein; LDL=low-density lipoprotein; HDL=high-density lipoprotein; MUFA=monounsaturated fatty acids; PUFA=polyunsaturated fatty acids; VLDL=very-low-density lipoprotein. Note: Filled dot: CI do not include the null. MUFA, PUFA and saturated fatty acid concentrations include all fatty acids detected which have one, more than one, or zero C=C double bonds in their backbone, respectively.


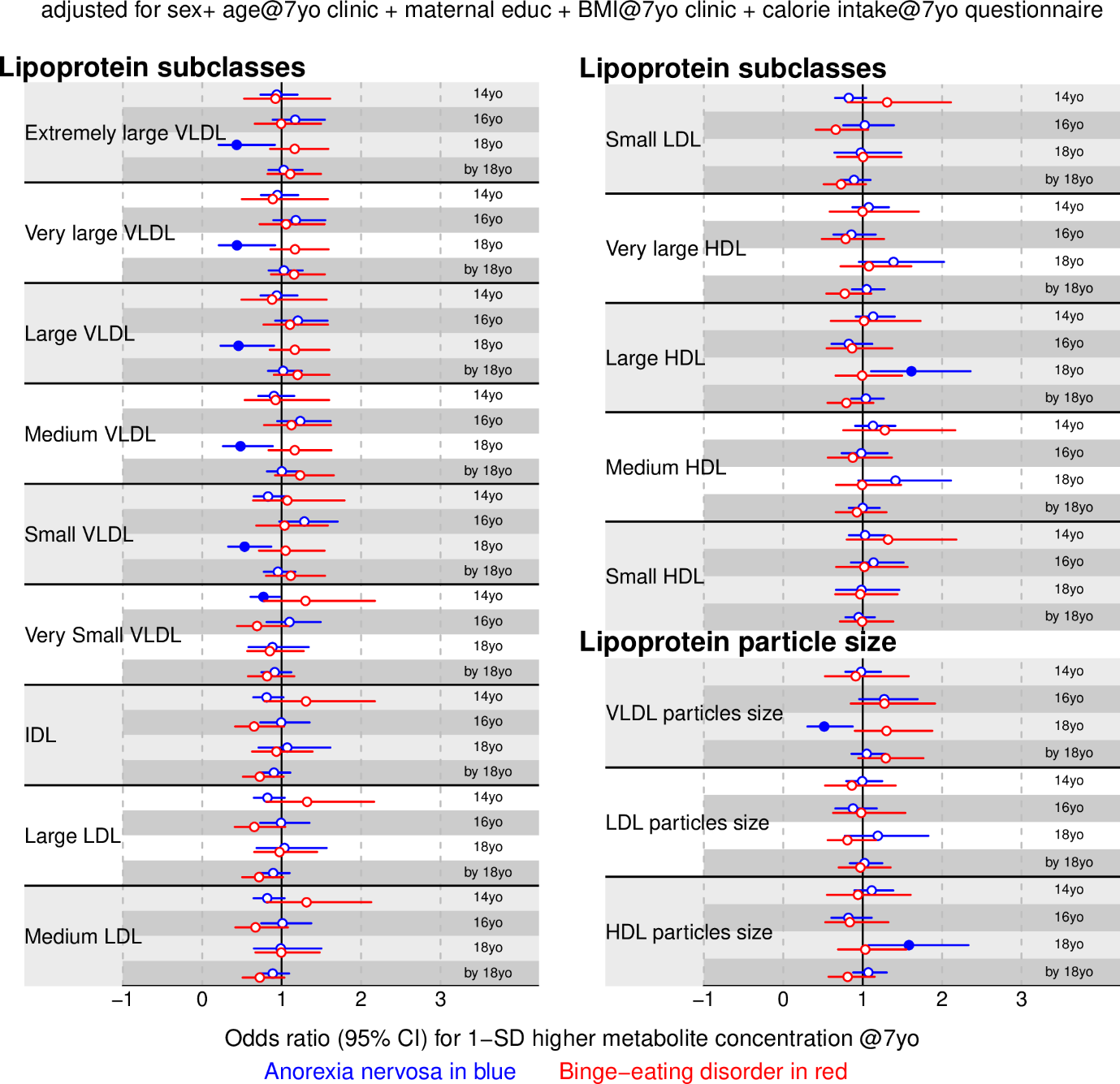


Figure S11b. Estimated odds ratios for binge-eating disorder (BED) and anorexia nervosa (AN) at 14, 16, 18 years of age and cumulatively across all three time points according to metabolic traits concentration at 7 years. Estimates refer to 1 standard deviation increase in metabolic trait concentration at 7 years. Error bars = 95% confidence intervals (CI). Abbreviations: C=cholesterol; IDL=intermediate-density lipoprotein; LDL=low-density lipoprotein; HDL=high-density lipoprotein; MUFA=monounsaturated fatty acids; PUFA=polyunsaturated fatty acids; VLDL=very-low-density lipoprotein. Note: Filled dot: CI do not include the null. MUFA, PUFA and saturated fatty acid concentrations include all fatty acids detected which have one, more than one, or zero C=C double bonds in their backbone, respectively.


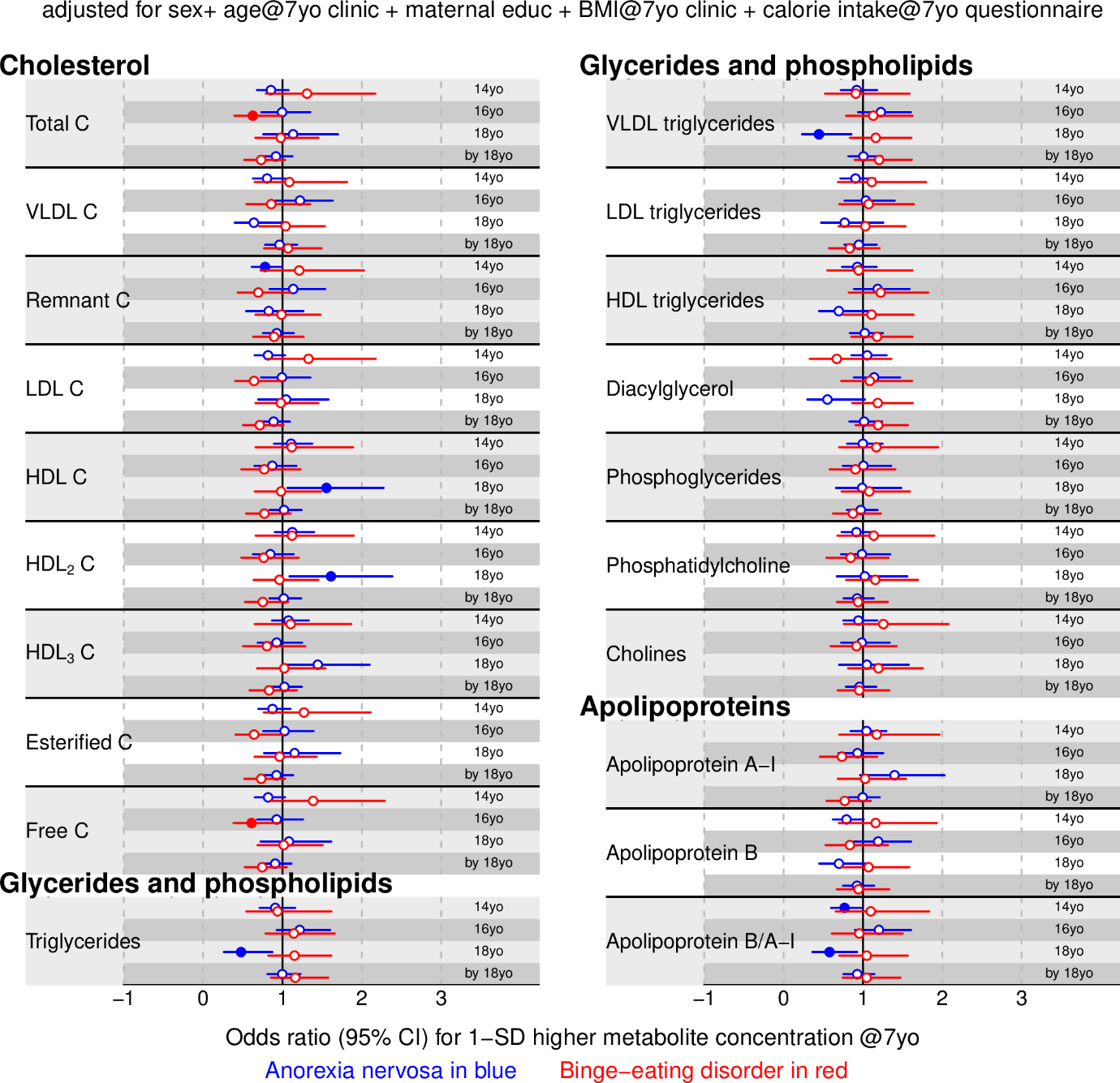


Figure S11c. Odds Ratios for binge-eating disorder (BED) and anorexia nervosa (AN) at 14, 16, 18 years of age and cumulatively across all three time points according to metabolic traits concentration at 7 years. Estimates refer to 1 standard deviation increase in metabolic trait concentration at 7 years. Error bars = 95% confidence intervals (CI). Abbreviations: C=cholesterol; IDL=intermediate-density lipoprotein; LDL=low-density lipoprotein; HDL=high-density lipoprotein; MUFA=monounsaturated fatty acids; PUFA=polyunsaturated fatty acids; VLDL=very-low-density lipoprotein. Note: Filled dot: CI do not include the null. MUFA, PUFA and saturated fatty acid concentrations include all fatty acids detected which have one, more than one, or zero C=C double bonds in their backbone, respectively.


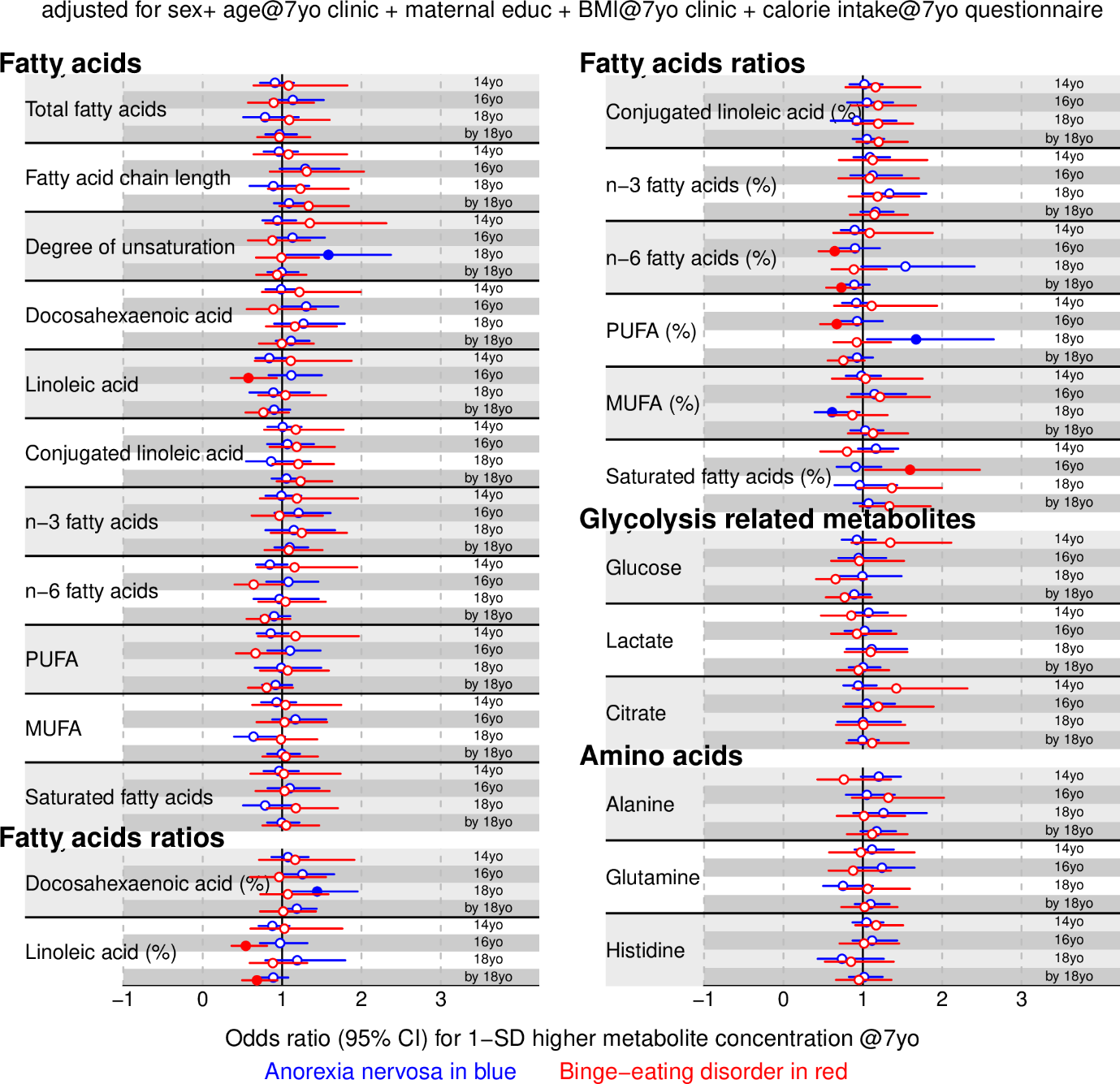


Figure S11d. Odds Ratios for binge-eating disorder (BED) and anorexia nervosa (AN) at 14, 16, 18 years of age and cumulatively across all three time points according to metabolic traits concentration at 7 years. Odds Ratios for BED and AN in adolescence reflect the risk per standard deviation increase in metabolic trait concentration at 7 years. Error bars = 95% confidence intervals (CI). Abbreviations: C=cholesterol; IDL=intermediate-density lipoprotein; LDL=low-density lipoprotein; HDL=high-density lipoprotein; MUFA=monounsaturated fatty acids; PUFA=polyunsaturated fatty acids; VLDL=very-low-density lipoprotein. Note: Filled dot: CI do not include the null. MUFA, PUFA and saturated fatty acid concentrations include all fatty acids detected which have one, more than one, or zero C=C double bonds in their backbone, respectively.


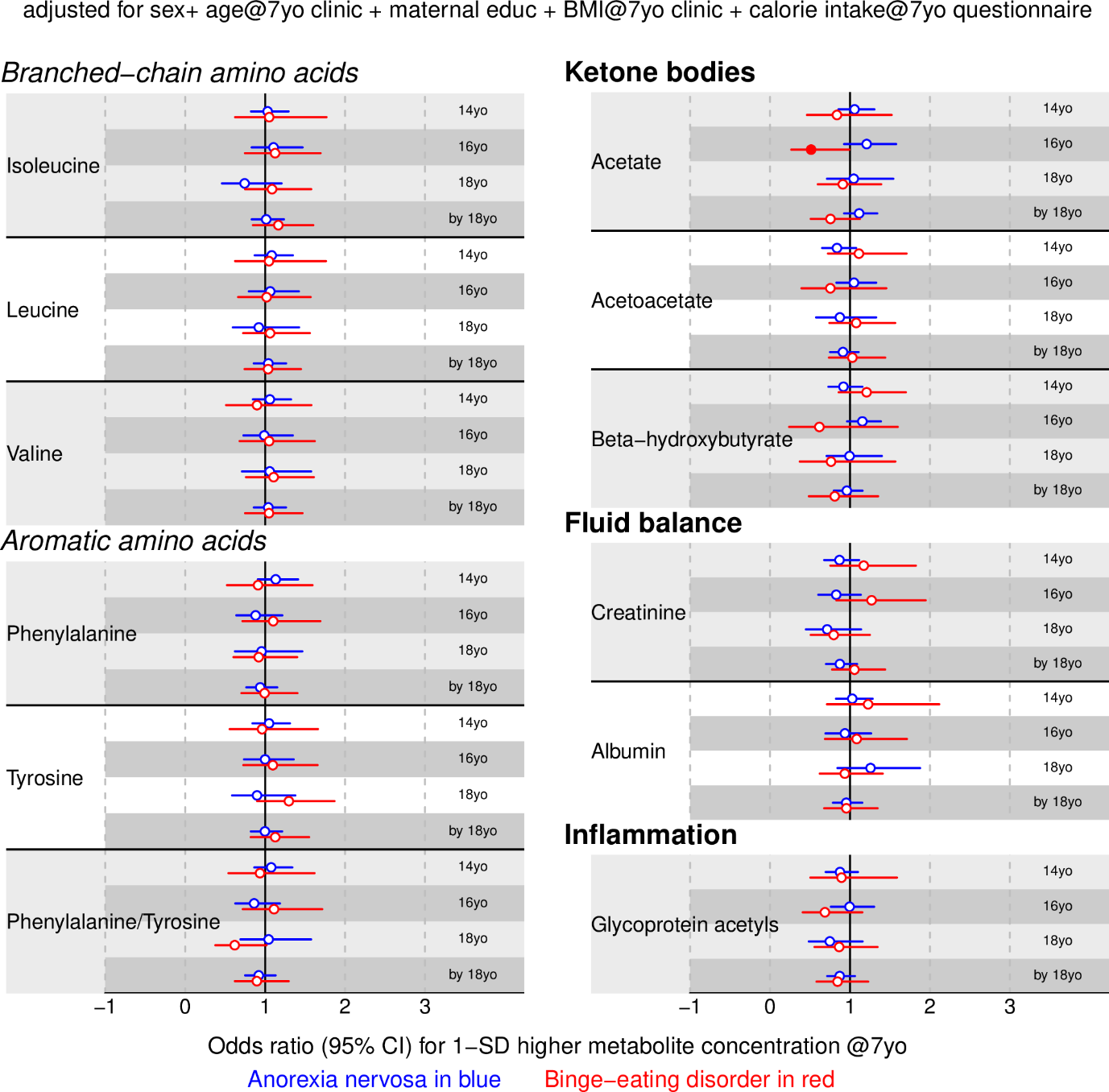


# Supplementary tables

Table S1. Distribution of background characteristics according to follow-up information available in the Avon Longitudinal Study of Parents and Children (ALSPAC) cohort.

| Background characteristics | All offspring in ALSPAC (*n*=19,290) | Offspring that attended 7 years clinic (*n*= 8,293) | Offspring included in the analysis  (*n*= 2,929) |
| --- | --- | --- | --- |
| **Female [n (%)]** | 9316 (48) | 4088 (49) | 1492 (51) |
| **Maternal education** |  |  |  |
| *>12 years in education [n (%)]* | 4394 (23) | 3218 (39) | 1459 (50) |
| *Missing* | 6872 (36) | 758 (9) | 0 |
| ***Age (years) [mean (SD)] at 7 years clinic*** |  | 7.5 (0.3) | 7.5 (0.1) |
| ***Body mass index (kg/m^2^) [mean (SD)] at 7 years clinic*** |  | 16.3 (2.1) | 16 (1.8) |
